# Animal-Assisted Interventions Improve Mental, But Not Cognitive or Physiological Health Outcomes of Higher Education Students: a Systematic Review and Meta-analysis

**DOI:** 10.1007/s11469-022-00945-4

**Published:** 2022-11-15

**Authors:** Annalena Huber, Stefanie J. Klug, Annette Abraham, Erica Westenberg, Veronika Schmidt, Andrea S. Winkler

**Affiliations:** 1 Department of Sport and Health Sciences, Technical University of Munich, Munich, Germany; 2 Department of Neurology, Center for Global Health, Klinikum Rechts der Isar, Technical University of Munich, Munich, Germany; 3 Centre for Global Health, Institute of Health and Society, University of Oslo, Oslo, Norway

**Keywords:** Animal-assisted intervention, Higher education, Mental health, Systematic review, One Health

## Abstract

Due to the high burden of mental health issues among students at higher education institutions world-wide, animal-assisted interventions (AAIs) are being used to relieve student stress. The objective of this study was to systematically review of the effects of AAIs on the mental, physiological, and cognitive outcomes of higher education students. Randomized controlled trials using any unfamiliar animal as the sole intervention tool were included in this review. Study quality was assessed using the Cochrane Risk-of-Bias tool. Where possible, effect sizes (Hedges’ *g*) were pooled for individual outcomes using random-effects meta-analyses. Albatross plots were used to supplement the data synthesis. Of 2.494 identified studies, 35 were included. Almost all studies used dogs as the intervention animal. The quality of most included studies was rated as moderate. Studies showed an overall reduction of acute anxiety and stress. For other mental outcomes, studies showed smaller, but nonetheless beneficial effects. Studies showed no clear effect on physiological or cognitive outcomes. Strong methodological heterogeneity between studies limited the ability to draw clear conclusions.

**Supplementary Information:**

The online version contains supplementary material available at 10.1007/s11469-022-00945-4.

As highlighted by ongoing events such as climate change and the COVID-19 pandemic, which is strongly suspected to have zoonotic origins (Andersen et al., 2020), it is essential to acknowledge the interconnectedness of humans, animals, and the environment. This thought is at the core of the One Health concept, which aims to highlight the “synergistic benefit of closer cooperation between human, animal and environmental health sciences” (Amuasi et al., 2020). One example of a benefit derived from the connection between humans and animals is animal-assisted interventions (AAIs). Based on the definition presented by López-Cepero, in this review, AAIs are defined as any intervention that incorporates an element of human-animal interaction (HAI) with an unfamiliar animal, with the aim of improving a human health outcome (López-Cepero, 2020). Unfamiliar animals are defined as animals that are not owned by or living with participants. Most commonly AAIs use dogs as the intervention animal, but other animals such as cats, horses, birds, or fish are also sometimes used (Bert et al., 2016; Kamioka et al., 2014). Importantly, Howell et al. distinguish between visiting animals and therapy animals: while therapy animals are those who are included “in the work of a qualified health professional in the provision of […] treatment,” animals “that have suitable characteristics and are trained for public visitation by humans who volunteer to take them into facilities to bring enjoyment” are defined as visiting animals. Accordingly, in the context of higher education settings, we expect AAIs to mainly include visiting animals (Howell et al., 2022).

Past research has predominantly focused on the benefits of AAIs for clinical populations, and has found beneficial effects (Beetz et al., 2012; Maujean et al., 2015). Among autism and dementia patients, AAIs have been found to improve social interaction and reduce problematic behaviors such as aggression or agitation (Berry et al., 2013; O’Haire, 2013; W. Wood et al., 2017; Yakimicki et al., 2019). AAIs are especially beneficial for patients with mental disorders. Several systematic reviews have shown reductions of clinical symptoms of disorders like anxiety, depression, and schizophrenia, as well as improved engagement and social interaction (Brooks et al., 2018; Jones et al., 2019; Kamioka et al., 2014). In addition, AAIs have been shown to reduce stress and improve well-being among non-clinical populations (Ein et al., 2018; Kamioka et al., 2014; Nimer & Lundahl, 2007).

There is a particularly strong need for stress-reducing interventions among students at higher education institutions. A higher education institution is “any postsecondary institution of learning that usually affords, at the end of a course of study, a named degree, diploma, or certificate of higher studies” (*Higher Education*, 2020). Due to a multitude of factors including navigating a new environment, a high academic workload and financial pressures, the prevalence of stress, and symptoms of depression and anxiety disorders are worryingly high among higher education students worldwide (Bayram & Bilgel, 2008; Eisenberg et al., 2007). According to the Anxiety and Depression Association of America, for example, 85% of students feel overwhelmed by academic expectations and demands, over 40% of students state that anxiety is a top concern, and 30% of students state that stress negatively affects their academic performance (Anxiety & Depression Association of America, 2020; Austin et al., 2010). Similar results have been replicated among higher education students around the world (Bayram & Bilgel, 2008; Grützmacher et al., 2018; Mortier et al., 2018). The burden of mental health problems among students has been continuously increasing, and has been further exacerbated during the COVID-19 pandemic (Cao et al., 2020; Grützmacher et al., 2018; Son et al., 2020).

In light of these findings, AAIs are becoming increasingly common at higher education institutions to promote student mental health (Crossman & Kazdin, 2015). Such programs most commonly take the form of drop-in events where groups of students can freely interact with dogs and their handlers (Gee et al., 2017). AAIs in higher education settings are low-cost and easily scalable, allowing them to reach a large proportion of the student body (Bell, 2013; Crossman & Kazdin, 2015; Reynolds & Rabschutz, 2011; E. Wood et al., 2018). In addition, AAIs are not stigmatized like other traditional mental health services due to the overwhelmingly positive perception of AAIs among higher education students (Crossman & Kazdin, 2015). This makes AAIs an ideal universal intervention for mental health promotion efforts at higher education institutions (Greenberg & Abenavoli, 2017; Vadivel et al., 2021).

To confidently implement AAIs in higher education settings, a comprehensive overview of the current state of research is needed. Importantly, despite a growing number of studies assessing the efficacy of AAIs on students, there are only a limited number of published systematic reviews on the topic. Previous work on the effects of AAIs in educational settings includes a 2012 meta-analysis by Hummel and Randler (2012) and a 2017 systematic review by Brelsford et al. (2017), both of which focus on school-aged children. A recent systematic review by Parbery-Clark et al. is to the author’s knowledge, the first systematic review to focus on the effects of AAIs among higher education students (Parbery-Clark et al., 2021). However, Parbery-Clark et al. focus solely on mental health outcomes and exclude physiological and cognitive outcomes from their review. The objective of this systematic review was therefore to fill this gap in the literature by estimating the effects of AAIs in higher education settings on the mental, physiological, and cognitive outcomes of students. This review also aims to contribute evidence to the “shared medicines and interventions” subgroup of *The Lancet* One Health Commission (Amuasi et al., 2020).

## Methods

### Protocol and Registration

A systematic review protocol was developed in keeping with the PRISMA-P 2015 statement (Moher et al., 2015). This protocol was registered on PROSPERO on August 12, 2020, with the registration number CRD42020196283.

### Sources, Search Methods, and Eligibility Criteria

The literature search was conducted from June 10 to June 20, 2020, and was designed to identify all published and unpublished experimental and observational trials on AAIs conducted in higher education settings. Medline/PubMed, PsycInfo, CINAHL, Web of Science, Embase, ERIC, and Scopus were searched. In addition, WALTHAM Science, HABRI Central and Animal and Society Institute, and the database OpenGrey were searched. Reference lists from relevant systematic reviews and included studies were hand-searched for potentially relevant publications.

Due to the large number of retrieved results, only randomized controlled trials (RCTs) that were published in a peer-reviewed journal were included in this review. Studies were included if they assessed the effect of an AAI on any mental, physiological, or cognitive outcome of higher education students. Mental health outcomes were considered those that describe a person’s emotional or psychological state, for example, through self-perceived assessments of stress, anxiety, or depression. We also included physiological outcome measures that reliably correlate with acute stress, such as blood pressure (BP), heart rate (HR), or cortisol levels (APA, 2018). Cognitive outcomes were considered those that describe a person’s cognitive functioning (Henderson et al., 2015), for example, through assessments of intelligence, concentration, or attention. In the higher education context, we also considered cognitive outcomes to include academic outcomes such as test performance. Details on the eligibility criteria can be found in Table S1, while details on the search strategy can be found in File S1.

### Study Selection

The selection process was conducted in two steps, using Covidence (Covidence—Better Systematic Review Management, 2020). First, two independent reviewers (AH and EW) screened articles first by title and abstract, then by full text and voted on eligibility. Potential disagreements were resolved through regular discussions. If articles could not be found, the corresponding author was contacted. If there was no response within 2 weeks, the articles were excluded. Articles both reviewers agreed upon were included in the systematic review.

### Quality Assessment

Only quantitative outcomes that were assessed by at least three studies and could thus be meaningfully combined in a quantitative synthesis were included in the quality assessment process. The risk of bias of the included studies was assessed independently by two reviewers (AH and EW), using the Cochrane Risk-of-Bias tool for Randomized Trials 2 (RoB 2) (Sterne et al., 2019). The version for individually randomized, parallel-group trials and the version for crossover trials were used.

### Data Extraction

Data extraction was independently conducted by AH and EW using an Excel sheet. Data was collected on the study design, study participants, the intervention condition, the control condition, and reported outcomes. Conflicts were resolved through regular discussions. A full list of the extracted data items can be found in File S2.

### Data Synthesis

All studies were grouped according to the qualitative or quantitative outcomes they assessed. For stress, anxiety, and depression, we further differentiated between chronic (long-term) and acute (short-term) outcomes. We defined acute outcomes as measuring how a person is feeling in a given moment, and chronic outcomes as measuring how a person is feeling over a longer period of time.

#### Qualitative Synthesis

Quantitative outcomes reported by less than three studies, as well as all qualitative outcomes, were summarized in a qualitative synthesis. Study results were briefly summarized for each outcome. Studies assessing mental, physiological, and cognitive outcomes were grouped together, and common trends in results were described.

#### Quantitative Synthesis

Quantitative outcomes reported by three or more studies were included in the quantitative synthesis. For both the meta-analyses and the albatross plots, potential multiplicity was eliminated by applying the following rules: First, if an outcome was reported across multiple time-points, the last reported measurement of the outcome which was not yet part of follow-up measurements was chosen. Second, if an outcome was reported using multiple measures and the reported measures were assumed to be interchangeable, only one of the included measures was chosen. This was the case in studies reporting both systolic and diastolic BP, where systolic BP was chosen, and in studies reporting both HF (high-frequency) and rMSSD (root mean square of successive differences) heart rate variability (HRV), where HF HRV was chosen.

To be included in a meta-analysis, studies needed to supply an effect size (Hedges’ *g*) of the post-test difference in mental, physiological, or cognitive outcomes between an intervention and a control group, and had to be of good quality (rated as “low risk” or “some concerns” by the RoB 2). In addition, studies had to use comparable intervention and control conditions. Interventions generally fell into two categories: (1) interventions that allowed participants to freely interact with animals and their handlers (active intervention) and (2) interventions where an animal was present while participants’ primary focus was on a task (passive intervention). These tasks typically aimed to increase the stress levels of participants (stressors), such as timed math tasks. Interventions were additionally categorized based on the animal species used in the intervention condition. Control conditions broadly fell into four categories: (1) control groups that replaced the presence of an animal with a human (active human control); (2) control groups that replaced the presence of the animal with a different animal, a toy animal, or pictures/videos of an animal (active animal control); (3) control groups with an active component that was not a human or a different animal like yoga (active other control); and (4) control groups without any active component (no-treatment control). For each outcome included in the quantitative synthesis, coded tables were created to assess meta-analysis eligibility (Tables SIII–SXIV). Meta-analyses were conducted for all outcomes where at least three studies reported an effect size, were of good quality, and used comparable intervention and control conditions. Due to the small number of studies included in each meta-analysis, it was not possible to conduct moderator analyses.

For eligible outcomes, meta-analyses were conducted using RStudio Version 1.3.959 (RStudio Team, 2020). Summary effect sizes as well as the corresponding 95% confidence interval (CI) were calculated using a random-effects model, and visualized using forest plots. The heterogeneity between included studies was assessed using the *Q* and *I*^2^ statistics. If Hedges’ *g* and its standard error (SE) was not reported in the original study, it was computed in RStudio Version 1.3.959, using the package “esc” (Lüdecke, 2019). Details of the conducted calculations can be found in Table SXV. Funnel plots were used to explore publication bias, and Egger’s test for funnel plot asymmetry was conducted.

Due to the limited number of studies included in the meta-analyses, albatross plots were used to extend the quantitative data synthesis. The albatross plot is a graphical tool that allows an approximation of effect sizes based on p-value and sample size (Harrison et al., 2017). The eligibility criteria in place for the meta-analyses were not required for inclusion in the albatross plots. Albatross plots were created using Stata/SE 16.1 (Stata Statistical Software. College Station, TX: StataCorp LLC; 2019). Effect size contours were calculated based on the standardized mean difference (SMD). Contours corresponded to the effect sizes 0.2 (small effect), 0.5 (medium effect), and 0.8 (large effect). Since all included studies were randomized, an equal group size was assumed. As suggested by Harrison et al., if a *p*-value was presented as a threshold instead of an exact value (e.g., *p* < 0.05), the threshold value was used as the exact value (Harrison et al., 2017). In addition, for any non-significant outcome without an exact *p*-value (e.g., *p* > 0.05), a *p*-value of 1 was substituted (Harrison et al., 2017). If not reported in the original study, *p*-values were calculated by conducting unpaired two-sided Student’s *t*-tests in RStudio Version 1.3.959, using the command “t.test” and the mean, standard deviation, and sample size provided (RStudio Team, 2020). If not otherwise specified in the study, a normal distribution of the data was assumed.

The threshold for statistical significance was set at *p* < 0.05 for all conducted calculations. The code used for all calculations can be found under https://doi.org/10.6084/m9.figshare.19368047.v1.

## Results

### Study Selection

**Fig. 1:**
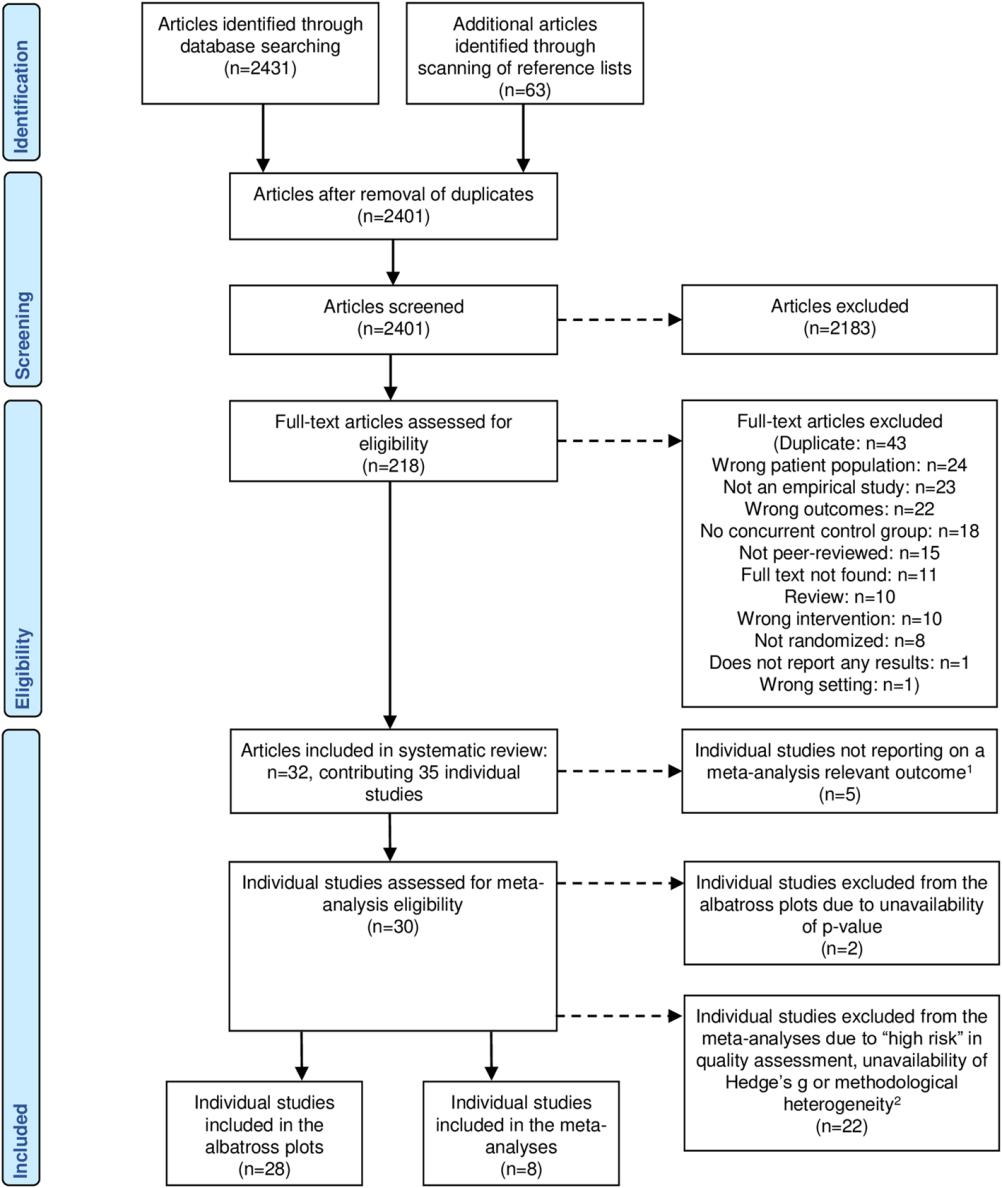
PRISMA flow chart. ^1^Quantitative outcomes assessed by less than three studies as well as qualitative outcomes were not eligible for inclusion in the meta-analyses. ^2^Methodological heterogeneity = heterogeneous for type of intervention condition (active/passive), animal used or type of control condition (active animal/active human/active other or no-treatment)

A search of all databases yielded 2.431 search results. Screening of reference lists contributed an additional 63 search results, giving a total of 2.494 results. Details on the exact number of results obtained from each database can be found in Table SII. After removing duplicates and screening the articles by title and abstract, a total of 218 articles remained for full text screening. After the full text screening, 32 articles remained for inclusion in this systematic review. Of these 32 articles, three reported two separate eligible studies (Crump & Derting, 2015; Gee et al., 2019; Trammell, 2017), bringing the total of individual studies included in this review to 35 (Banks et al., 2018; Barker et al., 2016, 2017; Binfet, 2017; Capparelli et al., 2020; Charnetski et al., 2004; Crossman et al., 2015; Crump & Derting, 2015; Fiocco & Hunse, 2017; Gebhart et al., 2020; Gee et al., 2014, 2015, 2019; González-Ramírez et al., 2016; Grajfoner et al., 2017; Hall, 2018; Hunt & Chizkov, 2014; Kobayashi et al., 2017; McDonald et al., 2017; Pendry et al., 2018, 2020; Pendry et al., 2019a; Pendry, Vandagriff, et al., 2019; Pendry & Vandagriff, 2019; Polheber & Matchock, 2014; Shearer et al., 2016; Stewart & Strickland, 2013; Straatman et al., 1997; Trammell, 2017, 2019; Ward-Griffin et al., 2018; Wilson, 1987). Common reasons for exclusion can be found in the PRISMA flow chart (Fig. 1). Of the 35 studies, 30 were included in the quantitative data synthesis. Of these, eight studies were included in the meta-analyses and 28 studies were included in the albatross plots.

### Study Characteristics

An overview of the most important extracted data items and study results can be found in the data extraction table (Table 1). Information on additional study characteristics can be found in Table SXVI. In general, most studies had more female than male participants, and participants were mostly of “typical” undergraduate age (mean 20.2 years, median 19.7 years). In almost all studies (*n* = 29), the intervention animal was a dog. While all studies used visiting animals as defined above, most intervention animals had received a therapy animal certification, while a small number of studies used companion animals (most commonly pets of researchers) who did not have a certification (Hunt & Chizkov, 2014; McDonald et al., 2017; Pendry et al., 2019a; Pendry, Vandagriff, et al 2019; Pendry & Vandagriff, 2019; Straatman et al., 1997). Most studies (*n* = 2 7) used active intervention conditions, with most taking place in a group setting. In studies with active interventions, the animal-to-participant ratio was generally 1:3–5 participants. The remaining studies (*n* = 8) used passive intervention conditions that mostly took place in individual settings and included a stressor. In studies with passive interventions, the animal-to-participant ratio was generally 1:1. The most common control condition was a no-treatment control condition (*n* = 27). In most studies (*n* = 28), intervention sessions took place only once per participant. In general, intervention sessions were relatively short (mean 20.7 min, median 15 min).

**Table 1:** Data extraction table (n = 35)

Animal	Date	Reference	Design	N	Results of studies included in qualitative synthesis^a,b^	Direction of effect^c,d^	Results of studies included in quantitative synthesis^a,b^	Direction of effect^c,d^
**Dog**	2020	Capparelli (Capparelli et al., 2020)	RCT	64	N/A	N/A	Passive intervention vs no treatment control: Performance on a memory task: p = 0.8	Null
Dog	2020	Pendry (Pendry et al., 2020)	RCT	309	Active intervention vs other control (alternative stress management techniques only):		N/A	N/A
					WILL-subscale: p = 0.021	Positive		
					SELFREGULATION-subscale: p = 0.215	Null		
					SKILL-subscale: p > 0.05	Null		
					Active intervention vs other control (human-animal interaction and alternative stress management techniques):			
					WILL-subscale: p > 0.05	Null		
					SELFREGULATION-subscale: N/A	N/A		
					SKILL-subscale: p > 0.05	Null		
Dog	2019	Gebhart (Gebhart et al., 2020)	RCT	57	Active intervention vs no treatment control (no exam): IgA: N/A Active intervention vs no treatment control (exam): IgA: N/A Active intervention vs other control: IgA: N/A	N/A N/A N/A	Active intervention vs no treatment control (no exam): Acute self-perceived stress: p < 0.01 Acute anxiety: p < 0.01 Salivary cortisol: N/A Active intervention vs no treatment control (exam): Acute self-perceived stress: p > 0.5 Acute anxiety: p > 0.5 Salivary cortisol: N/A Active intervention vs other control: Acute self-perceived stress: N/A Acute anxiety: N/A Salivary cortisol: N/A	Negative Negative N/A Null Null N/A N/A N/A N/A
Dog	2019	Pendry (Pendry, Kuzara, et al., 2019)	RCT	228	Active intervention vs other controls (alternative stress management techniques only; human-animal interaction and alternative stress management techniques): Behaviour change: p = 0.31 Changes in home-practice: p = 0.44 Qualitative interviews revealed students' favourite part of dog interaction was positive effect on mood	Null Null Positive	N/A	N/A
Dog	2019	Trammell (Trammell, 2019)	RCT	44	N/A	N/A	Passive intervention vs no treatment control: Acute self-perceived stress: p = 0.03 Arousal: p > 0.05 Happiness: p > 0.001 Performance on a memory task: p = 0.34	Negative Null Positive Negative
Dog	2018	Banks (Banks et al., 2018)	RCT	56	Active intervention vs no treatment control: Cognitive test anxiety: p = 0.62 Concentration: p > 0.05	Null Null	Active intervention vs no treatment control: Acute anxiety: p = 0.023 Negative affect: p = 0.84 Chronic self-perceived stress: p = 0.042 Positive affect: p = 0.97	Negative Null Negative Null
Dog	2018	Hall (Hall, 2018)	RCT	98	N/A	N/A	Active intervention vs no treatment control: Acute anxiety: p = 0.008	Negative
Dog	2018	Ward-Griffin (Ward-Griffin et al., 2018)	RCT	246	Active intervention vs no treatment control: Social support: p = 0.25 Life satisfaction: p = 0.87	Null Null	Active intervention vs no treatment control: Negative affect: p = 0.5 Chronic self-perceived stress: p = 0.95 Happiness: p = 0.89 Positive affect: p = 0.76	Negative Null Null Null
Dog	2017	Barker (Barker et al., 2017)	RCT	74	Active intervention vs no treatment control: Distance between self and family members: p = 0.562 Distance to personal stressors: p = 0.02 Distance to other stressors: p = 0.05	Null Negative Null	N/A	N/A
Dog	2017	Binfet (Binfet, 2017)	RCT	163	Active intervention vs no treatment control: Homesickness: p < 0.05 Sense of belonging in school: p = 0.002	Negative Positive	Active intervention vs no treatment control: Chronic self-perceived stress: p = 0.019^e^	Negative
Dog	2017	Fiocco & Hunse (Fiocco & Hunse, 2017)	RCT	61	Active intervention vs no treatment control: Galvanic skin response: N/A	N/A	Active intervention vs no treatment control: Negative affect: N/A positive affect: N/A	N/A
Dog	2017	Grajfoner (Grajfoner et al., 2017)	RCT	132	Active intervention vs human control vs animal control: Mood: p = 0.011 Well-being: p < 0.001	Negative (higher in animal control than other conditions) Positive (higher in intervention and animal control compared to no treatment control)	Active intervention vs human control: Acute anxiety: p < 0.001 Active intervention vs animal control: Acute anxiety: p < 0.001	Negative Positive
Dog	2017	McDonald (McDonald et al., 2017)	RCT	48	N/A	N/A	Active intervention vs no treatment control: BP: p < 0.001	Negative
Dog	2017	Trammell—Study 2 (Trammell, 2017)	RCT	44	Active intervention vs animal control: Study effort for final exam: p > 0.43	Null	Active intervention vs animal control: Acute self-perceived stress: p = 0.02 Performance on a memory task: p = 0.62	Negative Null
Dog	2017	Trammell—Study 3 (Trammell, 2017)	RCT	45	Active intervention vs animal control: Study effort for final exam: p > 0.10	Null	Active intervention vs animal control: Acute self-perceived stress: p = 0.30 Performance on a memory task: p = 0.67	Negative Null
Dog	2016	Barker (Barker et al., 2016)	cRCT	57	Active intervention vs no treatment control: sAA: p = 0.356 sNGF: dropped from analysis	Null N/A	Active intervention vs no treatment control: Acute self-perceived stress: p = 0.0001	Negative
Dog	2016	González-Ramirez (González-Ramírez et al., 2016)	RCT	14	Active intervention vs no treatment control: Stress caused by public speaking: p > 0.05 Stress management: p = 0.805	Null Null	N/A	N/A
Dog	2015	Crossman (Crossman et al., 2015)	RCT	67	N/A	N/A	Active intervention vs no treatment control: Acute anxiety: p < 0.0001^e^ Negative affect: p < 0.0001^e^ Positive affect: p = 0.076 Active intervention vs animal control: Acute anxiety: p < 0.0001^e^ Negative affect: p < 0.0001^e^ Positive affect: p = 0.1	Negative Negative Positive Negative Negative Positive
Dog	2015	Crump—Study I (Crump & Derting, 2015)	cRCT	27	N/A	N/A	Active intervention vs no treatment control: Acute self-perceived stress: p = 0.029 Arousal: p = 0.006 HR: p > 0.05 BP: p = 0.039	Negative Positive Null Positive
Dog	2015	Crump—Study II (Crump & Derting, 2015)	RCT	61	N/A	N/A	Active intervention vs no treatment control: Acute self-perceived stress: p = 0.046 Chronic self-perceived stress: p > 0.05 Arousal: p = 0.007 Salivary cortisol: p > 0.05	Negative Null Positive Null
Dog	2015	Gee (Gee et al., 2015)	cRCT	31	N/A	N/A	Passive intervention vs no treatment control vs animal control vs human control: HR: p = 0.55 HRV: p = 0.49 Passive intervention (touch) vs no treatment control vs human control: Performance on a memory task: p < 0.05 Passive intervention (no touch) vs no treatment control vs human control: Performance on a memory task: p > 0.05	Null Positive Negative Null
Dog	2015	Shearer (Shearer et al., 2016)	RCT	74	Active intervention vs no treatment control vs other control: Mindfulness: p > 0.5 Active intervention vs no treatment control vs other control: Chronic depression: p > 0.05	Null Null	Active intervention vs no treatment control: Acute anxiety: p < 0.05 Negative affect: p = 0.001 HRV: p > 0.05 Active intervention vs other control: Acute anxiety: p < 0.05 Negative affect: p > 0.05 HRV: p < 0.05	Negative Negative Null Positive Null Negative
Dog	2014	Gee (Gee et al., 2014)	cRCT	53	N/A	N/A	Passive intervention vs animal control vs human control: HR: p = 0.046 HRV: p = 0.88 Performance on a memory task: N/A	N/A Null N/A
Dog	2014	Hunt & Chizkov (Hunt & Chizkov, 2014)	RCT	107	Passive intervention vs no treatment control: Chronic depression: p < 0.05	Negative	Passive intervention vs no treatment control: Negative affect: N/A Acute anxiety: N/A	N/A N/A
Dog	2014	Polheber & Matchock (Polheber & Matchock, 2014)	RCT	48	N/A	N/A	Passive intervention vs no treatment control vs human control: Acute anxiety: p > 0.05 HR: p > 0.5 Passive intervention vs no treatment control: Salivary cortisol: p = 0.024 Passive intervention vs human control: Salivary cortisol: p = 0.045	Null Null Negative Negative
Dog	2013	Stewart & Strickland (Stewart & Strickland, 2013)	RCT	128	N/A	N/A	Passive intervention vs no treatment control: Acute anxiety: p = 0.09	Negative
Dog	2004	Charnetski (Charnetski et al., 2004)	RCT	55	Active intervention vs no treatment control vs animal control: IgA: p > 0.05	Null	N/A	N/A
Dog	1997	Straatman (Straatman et al., 1997)	RCT	36	Passive intervention vs no treatment control: MAP: p = 0.0338	Null	Passive intervention vs no treatment control: HR: p = 0.338 BP: p = 0.338 Acute anxiety: N/A	Negative Positive N/A
Dog	1987	Wilson (Wilson, 1987)	cRCT	92	Active intervention vs no treatment control: MAP: p = 0.0005 Chronic anxiety: p = 0.761 Active intervention vs other control: MAP: p = 0.0005 Chronic anxiety: p = 0.317	Positive Null Negative Null	Active intervention vs no treatment control: Acute anxiety: p = 0.937 HR: p = 0.001 BP: p = 0.0005 Active intervention vs other control: Acute anxiety: p = 0.0005 HR: p = 0.0005 BP: p = 0.0005	Null Positive Positive Negative Negative Negative
**Dogs, cats**	2019	Pendry & Vandagriff (Pendry & Vandagriff, 2019)	RCT	249	N/A	N/A	Active intervention vs no treatment control: Salivary cortisol: p = 0.033 Active intervention vs animal control (slideshow): Salivary cortisol: p = 0.046 Active intervention vs animal control (observation): Salivary cortisol: p = 0.04	Negative Negative Negative
Dogs, cats	2019	Pendry (Clinical depression) (Pendry, Vandagriff, et al., 2019)	RCT	192	Active intervention vs no treatment control vs animal control: Irritability: N/A Contentness: N/A Acute depression: N/A Chronic depression: N/A	N/A	Active intervention vs no treatment control vs animal control: Acute anxiety: N/A	N/A
Dogs, cats	2018	Pendry (Pendry et al., 2018)	RCT	182	Active intervention vs animal control: Contentness: p < 0.01 Irritability: p = 0.03 Acute depression: p > 0.05 Active intervention vs no treatment control: Contentness: p < 0.001 Irritability: p = 0.02 Acute depression: p > 0.05	Positive Negative Null Positive Negative Null	Active intervention vs animal control: Acute anxiety: p < 0.01 Active intervention vs no treatment control: Acute anxiety: p = 0.03	Negative Negative
**Fish**	2019	Gee—Experiment 1 (Gee et al., 2019)	cRCT	35	Active intervention vs no treatment control: Relaxation: p = 0.001 Active intervention vs animal control: Relaxation: p < 0.001	Positive Positive	Active intervention vs no treatment control: Happiness: p < 0.001 HR: p > 0.05 Active intervention vs animal control: Happiness: N/A HR: p = 0.006 Active intervention vs no treatment control vs animal control: HRV: p > 0.05	Positive Null N/A Negative Null
Fish	2019	Gee—Experiment 2 (Gee et al., 2019)	RCT	39	Active intervention vs no treatment control: Relaxation: p = 0.001 Active intervention vs animal control: Relaxation: N/A	Positive N/A	Active intervention vs no treatment control: Acute anxiety: p < 0.001 Active intervention vs animal control: Acute anxiety: N/A Active intervention vs no treatment control vs animal control: HR: p > 0.05 Active intervention vs no treatment control vs animal control: HRV: p > 0.05	Negative N/A Null Null
**Cat**	2017	Kobayashi (Kobayashi et al., 2017)	cRCT	30	Active intervention vs animal control: Levels of oxygenated hemoglobin in prefrontal cortex: N/A Pleasure: p < 0.0001	N/A Positive	Active intervention vs no treatment control: Arousal: p > 0.05	Null

cRCT: crossover RCT. N/A: not applicable (information not given, or no p-value or direction of effect available/calculatable). N: number of participants. ^a^Abbreviations for outcomes: Blood pressure (BP), heart rate (HR), heart rate variability (HRV), mean arterial pressure (MAP), salivary nerve growth factor (sNGF), salivary alpha amylase (sAA), Immunoglobulin A (IgA). ^b^Results for main effect of condition at post-test. ^c^Negative direction of effect: levels of outcome were lower in intervention compared to the control group. Positive direction of effect: levels of outcome were higher in intervention compared to control group. ^d^Effect sizes were described as “null”, indicating no effect of the intervention on the corresponding outcome, if the p-value corresponding to the effect size was non-significant and the effect size was very small (less than 0.2). If the effect size was above 0.2, the direction of effect was specified even if the associated p-value was non-significant. ^e^p-value calculated by AH.

Outcomes were grouped into mental health outcomes, physiological outcomes, and cognitive outcomes. Mental health outcomes were by far the most common (*n* = 26), followed by physiological outcomes (*n* = 14), and cognitive outcomes (*n* = 9). Most reported cognitive outcomes were related to students’ academic performance.

### Risk of Bias Within Studies

Thirty studies were included in the quality assessment. Overall, 60 outcomes from 27 studies were classed as “some concerns,” 6 outcomes from 5 studies were classed as “high risk,” and no studies were classed as “low risk.” Common limitations included not reporting the method of allocation sequence generation or allocation sequence concealment. Additionally, blinding of participants and study personnel to a participants’ allocated condition was generally not possible due to the animal presence, although some studies tried to conceal the true study purpose from participants. Nonetheless, in most studies, both participants and study personnel were probably aware of their assigned condition, which may have especially affected self-reported outcomes. Finally, none of the included crossover RCTs gave information about potential carryover effects. An overview of quality assessment results for RCTs and crossover RCTs can be found in Figures S1 and S2. Quality assessment results at the individual outcome level can be found in Tables SXVII and SXVIII.

### Synthesis of Results

#### Qualitative Synthesis

Consistent with the study hypotheses, most studies reporting on negative mental health outcomes, including acute depression, chronic depression, homesickness, and irritability, reported lower levels of these outcomes in the intervention group compared to the control group at post-test (Binfet, 2017; Hunt & Chizkov, 2014; Pendry et al., 2018; Pendry, Vandagriff, et al., 2019, Wilson, 1987). Only Wilson et al. did not report an effect of the intervention on chronic anxiety (Wilson, 1987), and Shearer et al. did not report an effect on chronic depression (Shearer et al., 2016). Similarly, some studies reporting on positive mental health outcomes reported higher levels of these outcomes in the intervention group compared to the control group at post-test (Barker et al., 2017; Binfet, 2017; Gee et al., 2019; Grajfoner et al., 2017; Kobayashi et al., 2017; Pendry et al., 2018; Pendry et al., 2019a; Pendry, Vandagriff, et al., 2019). However, a few studies also reported no effect of the intervention on positive mental health outcomes, including mood, life satisfaction, and mindfulness (Barker et al., 2017; Grajfoner et al., 2017; Shearer et al., 2016; Ward-Griffin et al., 2018). Most studies reporting on physiological outcomes reported no effect of the intervention (Barker et al., 2016; Charnetski et al., 2004; Straatman et al., 1997), although Fiocco and Hunse reported a smaller electrodermal response after a stressor in the intervention compared to the control group (Fiocco & Hunse, 2017), and Wilson et al. found mean arterial pressure (MAP) to be significantly higher in the intervention compared to the control condition (Wilson, 1987). Similarly, most studies reporting on cognitive outcomes showed no effect of the intervention (Banks et al., 2018; González-Ramírez et al., 2016; Pendry et al., 2020; Pendry et al., 2019a). Only Pendry et al. found an improvement in test anxiety, attitude and study motivation (Pendry et al., 2020) (Table 1).

#### Quantitative Synthesis

The following outcomes were included in the quantitative synthesis: acute self-perceived stress, chronic self-perceived stress, negative affect, acute anxiety, arousal, happiness, positive affect, BP, HR, HRV, salivary cortisol, and performance on a memory task. Of these, meta-analyses were conducted for chronic self-perceived stress, negative affect, acute anxiety, positive affect, and BP. All studies included in the meta-analyses used an active intervention condition, a dog as the intervention animal and a no-treatment control condition. The most important results are presented below. Detailed results for the remaining outcomes, including the albatross plots and the meta-analyses, can be found in Figures S3–S13.

**Fig. 2:**
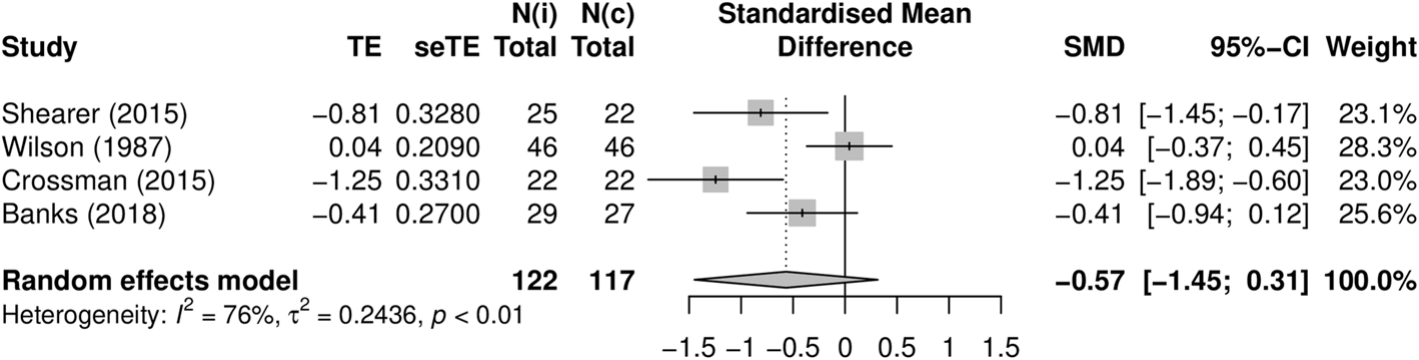
Forest plot acute anxiety (n = 4). TE: Hedges’ g. seTE: standard error of Hedges’ g. N(i): number of participants in intervention condition. N(c): number of participants in control condition

**Fig. 3:**
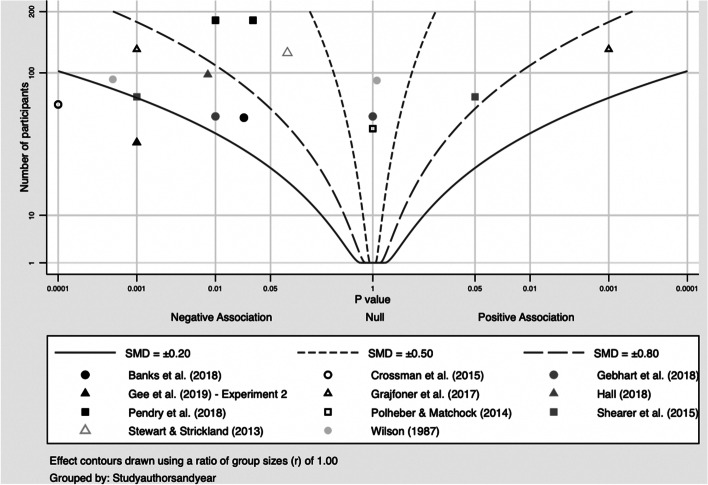
Albatross plot acute anxiety (n = 11)

**Fig. 4:**
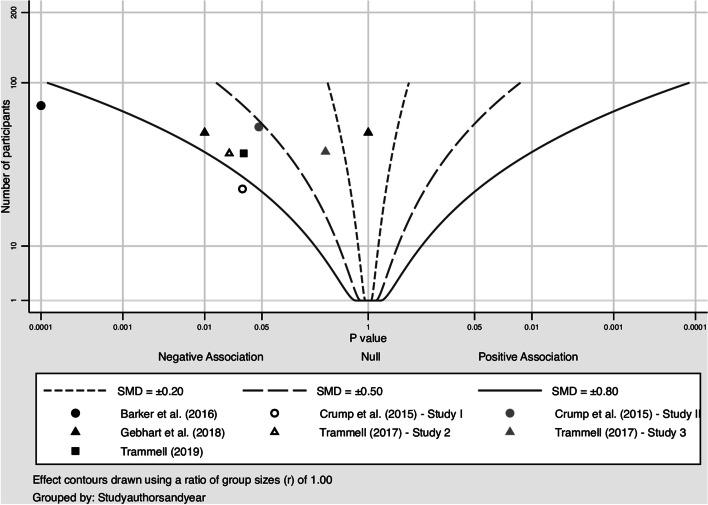
Albatross plot acute self-perceived stress (n = 7)

Mental health outcomes were most common. For acute anxiety and self-perceived stress, most included studies showed a clear reduction at post-test. Acute anxiety was reported by 14 studies, of which four studies were combined in a meta-analysis (Banks et al., 2018; Crossman et al., 2015; Shearer et al., 2016; Wilson, 1987). The pooled Hedges’ *g* was − 0.57 (95% CI: − 1.45, 0.31; *Q* = 12.5, *I*^2^ = 76%, *p* = 0.006), indicating a medium-sized negative effect of the intervention (Fig. 2). This result was mirrored by the albatross plot, where most studies clustered around the 0.5 to the 0.8 negative effect size contours (Fig. 3). Acute self-perceived stress was reported by seven studies. Although not combinable in a meta-analysis, the albatross plot demonstrated that included studies showed a reduction of self-perceived stress with a medium to large effect size, with most results clustering around the 0.5 to the 0.8 negative effect size contours of the albatross plot (Fig. 4).

**Fig. 5:**
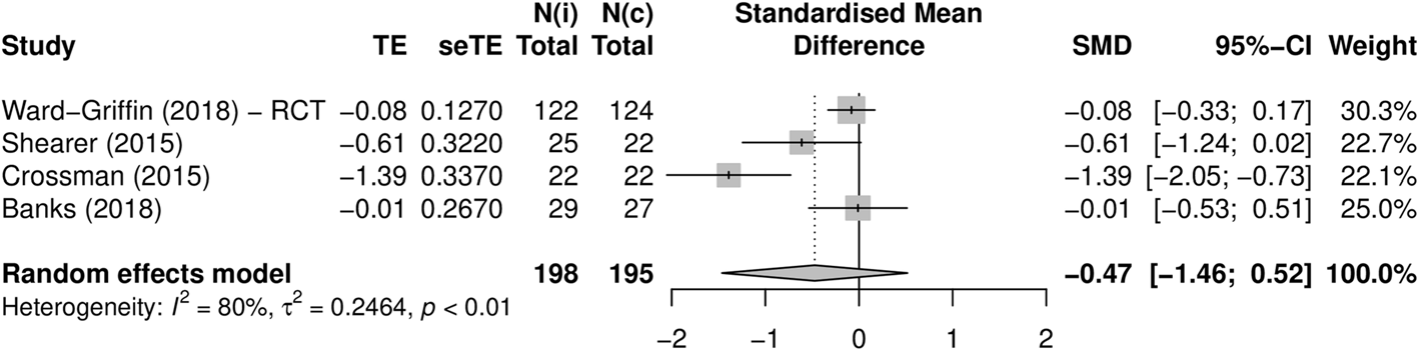
Forest plot negative affect (n = 4). TE: Hedges’ g. seTE: standard error of Hedges’ g. N(i): number of participants in intervention condition. N(c): number of participants in control condition

**Fig. 6:**
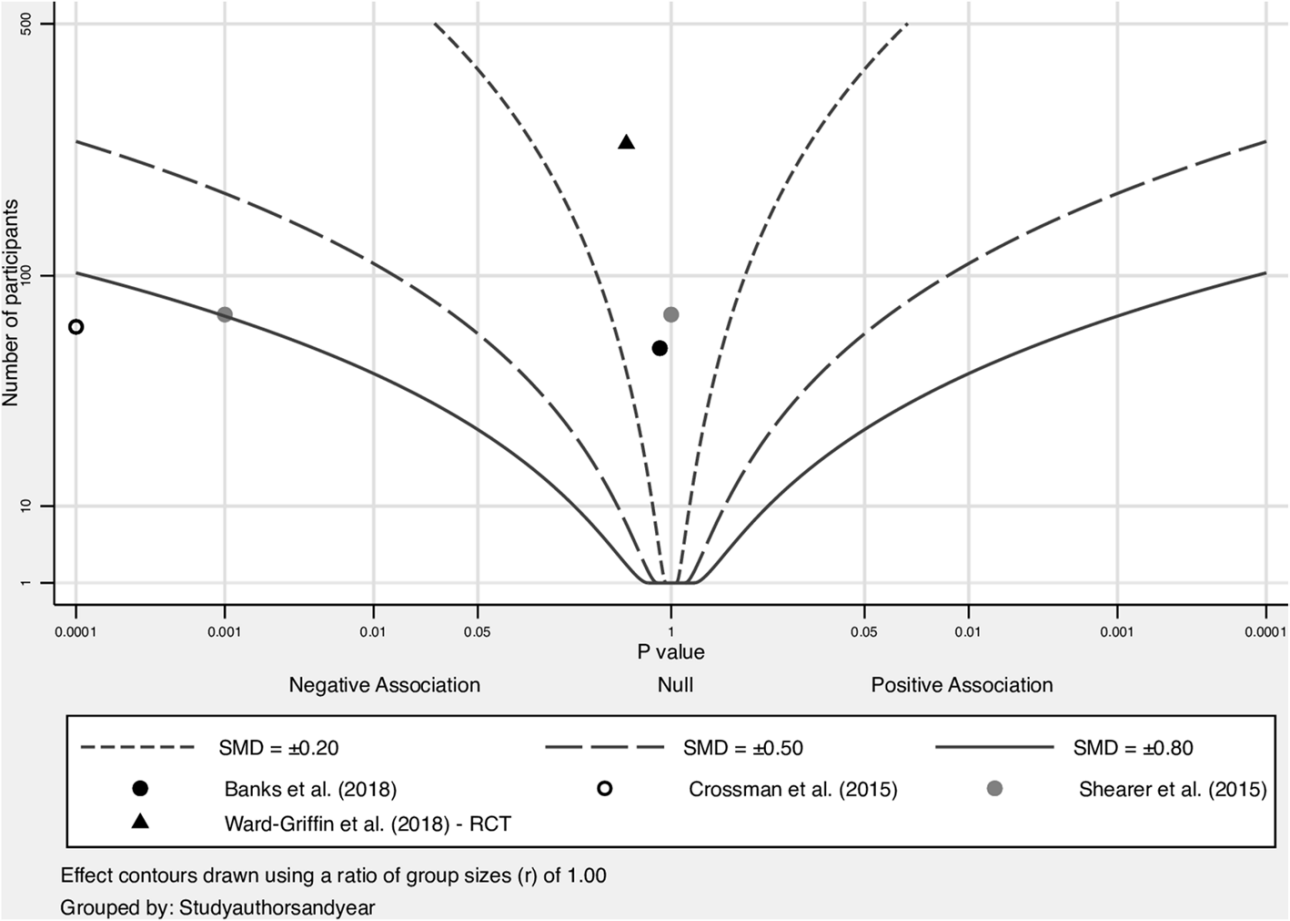
Albatross plot negative affect (n = 4)

Negative affect was reported by 6 studies, of which 4 studies were combined in a meta-analysis (Banks et al., 2018; Crossman et al., 2015; Shearer et al., 2016; Ward-Griffin et al., 2018). The pooled Hedges’ *g* was − 0.47 (95% CI: − 1.46, 0.52; *Q* = 15.3, *I*^2^ = 80.4%, *p* = 0.016), indicating a small- to medium-sized negative effect of the intervention (Fig. 5). The albatross plot showed that while some studies showed a reduction of negative affect, other studies showed no effect (Fig. 6). The tendency for some studies to show the expected effect while other studies showed no effect was also observed for the remaining mental health outcomes. Accordingly, a small negative effect of the intervention was observed for chronic self-perceived stress (pooled Hedges’ g: − 0.23 (95% CI: − 0.57, 0.11; heterogeneity: *Q* = 1.44, *I*^2^ = 0%, *p* = 0.49), and a small positive effect was observed for positive affect (pooled Hedges’ *g*: 0.06 (95% CI: − 0.78, 0.90; heterogeneity: *Q* = 3.97, *I*^2^ = 49.6%, *p* = 0.138), arousal, and happiness. Forest plots and albatross plots for these outcomes can be found in Figures S3–S8.

**Fig. 7:**
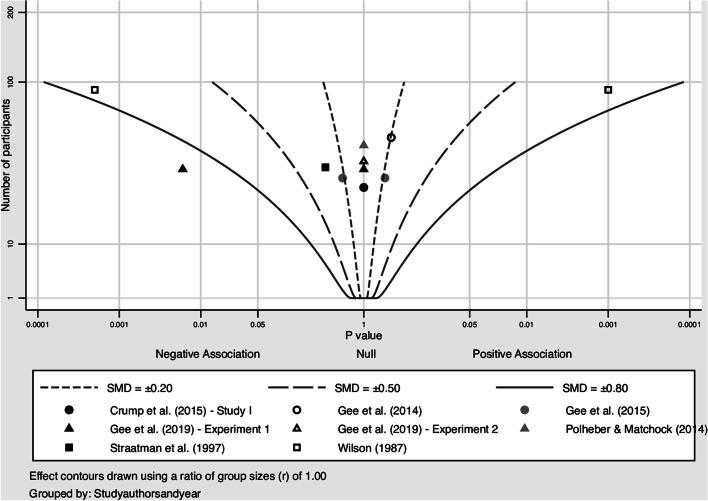
Albatross plot heart rate (n = 8)

Among the physiological outcomes, salivary cortisol was the only outcome to demonstrate the expected reduction post-intervention. Salivary cortisol was reported by four studies. Although not combinable in a meta-analysis, included studies showed a small to medium negative effect on cortisol, with most results falling between the 0.3 and 0.8 effect size contours of the albatross plot. In contrast, among the 8 studies assessing HR, most included studies showed no effect on HR, with most results clustered around the middle of the albatross plot (Fig. 7). This trend was mirrored by the studies assessing HRV.

Interestingly, studies reporting on BP showed very heterogeneous results. BP was reported by four studies, three of which were combinable in a meta-analysis. However, despite correcting for methodological heterogeneity, the results of studies included in the meta-analyses were very disparate in size and direction of effect. Additionally, the forest plot showed a very high, statistically significant level of heterogeneity between the included studies (*Q* = 45.5, *I*^2^ = 95.6%, *p* < 0.0001). These levels of heterogeneity were significantly higher than for any other meta-analysis conducted. Accordingly, it was deemed inappropriate to statistically combine BP outcomes, and no pooled effect size was calculated. This strong heterogeneity was mirrored in the albatross plot, where results were spread out throughout the plot. Forest and albatross plots for physiological outcomes can be found in Figures S9—S12.

The only cognitive outcome included in the quantitative synthesis was performance on a memory task, reported by six studies. Overall, included studies suggested a very small negative effect of the intervention on memory task performance, with most results clustering around the 0.2 effect size contour of the albatross plot. The albatross plot can be found in Figure S13.

### Risk of Bias Across Studies

The funnel plot showed no evidence of publication bias, as confirmed by Egger’s regression test for funnel plot asymmetry (*z* = − 1.74, *p* = 0.081). The funnel plot can be found in Figure S14.

## Discussion

### Summary of Findings

The aim of this systematic review was to assess the effect of AAIs implemented in higher education settings on the mental, physiological, and cognitive outcomes of students. In general, the results of this review suggest that AAIs in higher education settings are particularly effective at reducing acute feelings of anxiety and stress. The evidence is less clear for other mental health outcomes assessed in this review — the included studies suggest a smaller, but nonetheless beneficial effect of AAIs on these outcomes as well. This review does not suggest a clear beneficial effect of AAIs on physiological or cognitive outcomes of students. Overall, the quality of included studies was moderate, with most studies being classed as “some concerns”.

### Mental Health Outcomes

The beneficial effects on acute feelings of stress and anxiety are in keeping with previous systematic reviews, which have shown AAIs to improve these outcomes in a large variety of populations (Beetz et al., 2012; Bert et al., 2016; Ein et al., 2018). Parbery-Clark et al. also found included studies to have beneficial effects on self-perceived stress and anxiety among higher education students (Parbery-Clark et al., 2021). Similarly, previous reviews have shown AAIs to promote a positive mood, increase happiness, and reduce depressive symptoms (Beetz et al., 2012; Kamioka et al., 2014; Morrison, 2007; Souter & Miller, 2007). While the overall direction of effect of the included mental health outcomes was beneficial as expected, some studies reporting on mental health outcomes showed no effect of the intervention. Since formal moderator analyses were not possible in this review, we cannot say with certainty which, if any study characteristics are associated with this. The comparatively smaller effect sizes of chronic stress, anxiety, and depression, assessed with instruments designed to detect changes in the mental state over longer periods of time, may point to limited long-term effects of AAIs, as suggested in previous literature (Hillen, 2020; Serpell et al., 2017; Stern & Chur-Hansen, 2013).

Another possible contributor to differences in study results could be related to intervention design. Beetz et al. suggest that the beneficial effects of HAI could stem from an activation of the oxytocin (OT) system through sensory stimulation (Beetz et al., 2012). Specifically, they state that the closeness of the connection between human and animal, including the presence and duration of physical contact with the animal, is an important factor in if and how much OT is released during HAI (Beetz et al., 2012). Since participants in studies using a passive intervention and stressors were not able to focus completely on the present animal and often did not even touch the animal in question, it is possible that not enough OT was released to achieve the expected beneficial effects on mental health outcomes (Hunt & Chizkov, 2014; Stewart & Strickland, 2013). Since moderator analyses to confirm this hypothesis were not possible, future research could explore whether the use of passive interventions and stressors is indeed associated with a reduced effect of AAIs on the mental health outcomes of higher education students.

### Physiological Outcomes

Only three of the included studies provided results for cortisol, with two studies reporting reductions and one study reporting no effect on salivary cortisol at post-test (Crump & Derting, 2015; Pendry & Vandagriff, 2019; Polheber & Matchock, 2014). This trend towards a reduction of cortisol at post-test is in keeping with other literature (Beetz et al., 2012; Lundqvist et al., 2017). By contrast, studies assessing BP showed very mixed outcomes. This mixed effect of AAIs on BP has been reported in other systematic reviews, even though the overall trend suggests a decrease in BP post-AAI (Beetz et al., 2012; Bert et al., 2016). One possible explanation for the large discrepancies between BP results in this review and past reviews could be the poor reliability of BP as an outcome measure. Indeed, a study by Kelsey et al. showed that measures of cardiovascular reactivity, including BP, have a poor reliability across different typical stressor tasks (Kelsey et al., 2007). BP can be affected by variables such as the posture of the participant, movement or respiration (Kelsey et al., 2007). All of these factors differed between the studies included in this review. Additionally, while all studies used a BP monitor, measurements were taken from different locations including the upper arm (Crump & Derting, 2015; Wilson, 1987), the wrist (McDonald et al., 2017) or the finger (Straatman et al., 1997), which may also have contributed to the heterogeneity in results.

Interestingly, most included studies showed no effect of the intervention on HR or HRV. This is different from other reviews, which have found an overall reduction of HR after an AAI in a variety of populations (Bert et al., 2016; Ein et al., 2018). It is possible that AAIs may have less of an effect on physiological outcomes in young, healthy populations. Indeed, while Nimer and Lundahl found a significant improvement of physiological outcomes after an AAI, moderator analyses revealed that populations with disabilities showed significantly larger improvements than healthy populations (Nimer & Lundahl, 2007). Additionally, it is possible that differences in effects between studies are again associated with intervention design: Most studies that assessed HR and HRV included a stressor in their intervention, thus likely triggering an acute stress response among participants. It is well established that in response to an acute stressor, HR increases while HRV decreases (Chu et al., 2021). Accordingly, it is possible that in studies with a stressor, the potential effect of an AAI on these physiological outcomes was not strong enough to compete with or alter the effects of the acute stress response. More studies without an incorporated stressor would be needed to judge the effects of AAIs on the physiological outcomes of students in a non-stressful situation.

### Cognitive Outcomes

The studies included in this review showed no significant effect of the intervention on cognitive outcomes. This is an interesting finding, especially considering that past systematic reviews assessing the impact of AAIs among children have found that the presence of animals helped create a productive learning environment (Beetz et al., 2012; Brelsford et al., 2017). Although these systematic reviews point out that there is little evidence that AAIs directly improve academic performance, they have nevertheless been shown to improve related cognitive outcomes like concentration, motivation and attention (Beetz et al., 2012; Brelsford et al., 2017). However, Banks et al. hypothesized that while the presence of an animal may be beneficial for children, whose cognitive functions are still developing, there is less of an impact among higher education students, who are already at their peak of cognitive functioning (Banks et al., 2018). The primary benefits of AAIs for this population therefore seem to be affective, not cognitive.

### Limitations of Included Evidence

Included studies shared some characteristics that may limit the generalizability of review results. First, participants in the included studies were overwhelmingly female. This may be attributable to an increased interest in AAIs among females, as most studies recruited participants via self-selection, or to recruitment from traditionally majority-female degree programs, such as psychology or nursing (Fowler, 2018; US Bureau of Labor Statistics, 2020). Since previous research has shown differences between males and females in, for example, responses to stressors, it is possible that results may not be generalizable to both male and female students (Merz & Wolf, 2015; Taylor et al., 2014). Second, although the search strategy was designed to find publications using any intervention animal, almost all included studies used dogs. This may be due to the popularity of dogs as companion animals and the feelings of empathy and companionship associated with them, making them a popular choice for AAIs (Custance & Mayer, 2012). Additionally, dogs may have been the easiest option logistically since some studies cooperated with established university-based AAI programs that were already using dogs (Banks et al., 2018; Barker et al., 2016, 2017; Grajfoner et al., 2017; Pendry et al., 2018, 2020; Pendry et al., 2019a; Pendry, Vandagriff, et al., 2019; Pendry & Vandagriff, 2019; Trammell, 2017), and some studies used pet dogs of the researchers (Pendry & Vandagriff, 2019; Pendry et al., 2018; Pendry, Vandagriff, et al. 2019). Nonetheless, it should be kept in mind that the results of this review represent the effects of AAIs using dogs and are likely less applicable to AAIs using other animals. Other limitations of included studies were small sample sizes, lack of sample size calculations, and general lack of follow-up assessments.

### Limitations of the Review Process

The strength of this review lies in the use of the albatross plots to enrich the quantitative data synthesis, as well as the inclusion of RCTs only. Nonetheless, some important limitations remain.

First, the strong methodological heterogeneity severely limited the comparability of included studies, as has been the case with many other reviews in the AAI field (Bert et al., 2016; Brooks et al., 2018; Kamioka et al., 2014). The heterogeneity also limited the number of studies included in the individual meta-analyses and limited our ability to conduct moderator analyses. This heterogeneity is at least partly attributable to the broadly defined eligibility criteria used in this review. Kazdin et al. have remarked that such broad eligibility criteria, where inclusion is based on the presence of an animal in the intervention as opposed to the proposed mechanism of the intervention, is one of the reasons for the methodological heterogeneity in most reviews in the AAI field (Kazdin, 2017). This lack of a guiding theoretical framework in most reviews is exacerbated by the lack of an unanimously accepted theory on the mechanism of AAI effectiveness (Borrego et al., 2014). In order to limit this issue in future research, systematic reviews should settle on a specific theoretical framework to guide their eligibility criteria in order to include only logically comparable studies (Kazdin, 2017).

Second, the albatross plots in this review were explicitly meant to allow a more inclusive overview of available data than what was available based on meta-analyses alone, and were not meant to generate a usable summary statistic. The effect size contours superimposed on the plot are only approximations of the actual effect size (Harrison et al., 2017). While they allow a visual interpretation of the general trend of the included studies in terms of effect size and direction, they are not exact and are not to be interpreted as such (Harrison et al., 2017).

### Research Gaps and Implications

If possible, future reviews in this field could conduct moderator analyses to assess whether any study characteristics have an influence on study results. Future studies could explore whether incorporating a stressor in the study design or conducting an AAI in either a group or an individual setting influences the effect of AAIs on health outcomes. Additionally, while a recent review suggested that AAI participation has no adverse effects for participating animals, research is limited and results remain conflicting (Glenk, 2017). There is even less research on potential benefits of AAI participation for animals (Glenk, 2017). Interestingly, research has suggested that following stress, trauma, or abuse, animals can exhibit behavior similar to symptoms of human mental disorders such as depression or post-traumatic stress disorder (Ferdowsian et al., 2011; PTSD in Dogs, 2010). Taking this into account, it is essential that the physical and mental health of animals participating in AAIs is protected. In the best case, AAIs should be mutually beneficial to animals and humans, thus making them a truly shared intervention in the spirit of One Health.

One of the goals of this review was to provide an evidence base that administrators at higher education institutions can use to decide whether to implement AAIs at their own campus. Despite the methodological limitations listed, this review shows that AAIs can improve students’ mental health outcomes, especially acute feelings of anxiety and stress. Taking into consideration the high burden of mental health issues among students at higher education institutions, along with the unprecedented stress caused by the COVID-19 pandemic, higher education institutions will likely be facing an increasing demand for mental health support (Cao et al., 2020; Son et al., 2020; Vadivel et al., 2021). Due to their low cost, easy scalability and high popularity, AAIs present a good option for higher education institutions to improve student mental health (Reynolds & Rabschutz, 2011). This opportunity could be taken up particularly by universities outside of the US and Canada, where AAI programs are still rare. It has to be kept in mind, however, that while stress reduction efforts can certainly help, more structural changes should be implemented to reduce academic, social and financial pressures that impact students’ mental health. These could include an increased mental health budget at higher education institutions, reduced tuition fees, and a mandatory salary for student internships (Bayram & Bilgel, 2008; Hamaideh, 2011; Heckman et al., 2014).

## Conclusion

Overall, the results of this review suggest that AAIs in higher education settings can be effective at improving mental health outcomes of students and are particularly effective at reducing acute feelings of anxiety and stress. These findings have been replicated in many different settings and with a variety of populations. However, contrary to prior research, this review does not suggest a clear beneficial effect of AAIs on physiological or cognitive outcomes of students.

## Supplementary Information

Below is the link to the electronic supplementary material.

Supplementary file1 (DOCX 20 KB)

Supplementary Figure S1 (PDF 73 KB)

Supplementary Figure S2 (PDF 59 KB)

Supplementary Figure S3 (PDF 5 KB)

Supplementary Figure S4 (PDF 39 KB)

Supplementary Figure S5 (PDF 38 KB)

Supplementary Figure S6 (PDF 39 KB)

Supplementary Figure S7 (PDF 5 KB)

Supplementary Figure S8 (PDF 39 KB)

Supplementary Figure S9 (PDF 38 KB)

Supplementary Figure S10 (PDF 39 KB)

Supplementary Figure S11 (PDF 5 KB)

Supplementary Figure S12 (PDF 38 KB)

Supplementary Figure S13 (PDF 39 KB)

Supplementary Figure S14 (PDF 77 KB)

Supplementary File2 (PDF 121 KB)

Supplementary File3 (PDF 125 KB)

Supplementary Table S1 (PDF 53 KB)

Supplementary Table S2 (PDF 32 KB)

Supplementary Table S3 (PDF 75 KB)

Supplementary Table S4 (PDF 98 KB)

Supplementary Table S5 (PDF 77 KB)

Supplementary Table S6 (PDF 139 KB)

Supplementary Table S7 (PDF 73 KB)

Supplementary Table S8 (PDF 71 KB)

Supplementary Table S9 (PDF 77 KB)

Supplementary Table S10 (PDF 78 KB)

Supplementary Table S11 (PDF 123 KB)

Supplementary Table S12 (PDF 74 KB)

Supplementary Table S13 (PDF 72 KB)

Supplementary Table S14 (PDF 76 KB)

Supplementary Table S15 (PDF 73 KB)

Supplementary Table S16 (PDF 99 KB)

Supplementary Table S17 (PDF 92 KB)

Supplementary Table S18 (PDF 52 KB)

Supplementary Table S19 (PDF 61 KB)

## Data Availability

The datasets generated for this study can be found in the data repository Figshare under https://doi.org/10.6084/m9.figshare.19368047.v1. This manuscript was posted on the preprint server medRxiv under the title “Animals in higher education settings: Do animal-assisted interventions improve mental and cognitive health outcomes of students? A systematic review and meta-analysis” on April 16th, 2022, under the https://doi.org/10.1101/2022.04.11.22273607 (Huber et al., 2022).
