# Supplementary material for: Animal-Assisted Interventions Improve Mental, But Not Cognitive or Physiological Health Outcomes of Higher Education Students: a Systematic Review and Meta-analysis
Source: Int J Ment Health Addict. 2022 Nov 15:1–32. Online ahead of print. doi: 10.1007/s11469-022-00945-4 (PMC9666958; doi:10.1007/s11469-022-00945-4)
Supplement: Supplementary file 1 — Supplementary file1 (DOCX 20 KB) [file 11469_2022_945_MOESM1_ESM.docx]

**Supplementary material: Table of contents and figure captions**

File S1. Search strategy.

File S2. List of extracted data items.

Table SI. Eligibility criteria.

Table SII. Overview of search results.

Table SIII. Coded table for acute self-perceived stress (n=7).

# Table SIV. Coded table for chronic self-perceived stress (n=4).

# Table SV: Coded table for negative affect (n=4).

Table SVI: Coded table for acute anxiety (n=11).

# Table SVII: Coded table for arousal (n=4).

# Table SVIII: Coded table for happiness (n=3).

# Table SIX: Coded table for positive affect (n=3).

# Table SX: Coded table for blood pressure (n=4).

# Table SXI: Coded table for heart rate (n=8).

# Table SXII: Coded table for heart rate variability (n=5).

# Table SXIII: Coded table for salivary cortisol (n=3).

# Table SXIV: Coded table for performance on a memory task (n=5).

# Table SXV. Calculations for meta-analysis.

Table SXVI. Supplemental data extraction table.

# Table SXVII. Quality assessment results for RCTs at the individual outcome level (n(outcomes)=49).

# Table SXVIII. Quality assessment results for crossover RCTs at the individual outcome level (n(outcomes)=17).

Table SXIX. Completed PRISMA 2020 Checklist.

Figure S1. Overview of quality assessment results for RCTs (n(outcomes)=49). Results in %, absolute number of outcomes are inside the bars. Green corresponds to a rating of “low risk”, yellow to “some concerns”, and red to “high risk”.

Figure S2. Overview of quality assessment results for crossover RCTs (n(outcomes)=17). Results in %, absolute number of outcomes are inside the bars. Green corresponds to a rating of “low risk”, yellow to “some concerns”, and red to “high risk”.

Figure S3. Forest plot chronic self-perceived stress (n=3). TE: Hedges’ g. seTE: standard error of Hedges’ g. N(i): number of participants in intervention condition. N(c): number of participants in control condition.

Figure S4. Albatross plot chronic self-perceived stress (n=4).

Figure S5. Albatross plot arousal (n=4).

Figure S6. Albatross plot happiness (n=3).

Figure S7. Forest plot positive affect (n=3). TE: Hedges’ g. seTE: standard error of Hedges’ g. N(i): number of participants in intervention condition. N(c): number of participants in control condition.

Figure S8. Albatross plot positive affect (n=3).

Figure S9. Albatross plot heart rate variability (n=5).

Figure S10. Albatross plot salivary cortisol (n=3).

Figure S11. Forest plot blood pressure (n=3). TE: Hedges’ g. seTE: standard error of Hedges’ g. N(i): number of participants in intervention condition. N(c): number of participants in control condition.

Figure S12. Albatross plot blood pressure (n=4).

Figure S13. Albatross plot performance on a memory task (n=5).

Figure S14. Funnel plot (n=11).
