# Supplementary figures and images for: Animal-Assisted Interventions Improve Mental, But Not Cognitive or Physiological Health Outcomes of Higher Education Students: a Systematic Review and Meta-analysis
Source: Int J Ment Health Addict. 2022 Nov 15:1–32. Online ahead of print. doi: 10.1007/s11469-022-00945-4 (PMC9666958; doi:10.1007/s11469-022-00945-4)

### RCTs: Quality assessment results (in %)

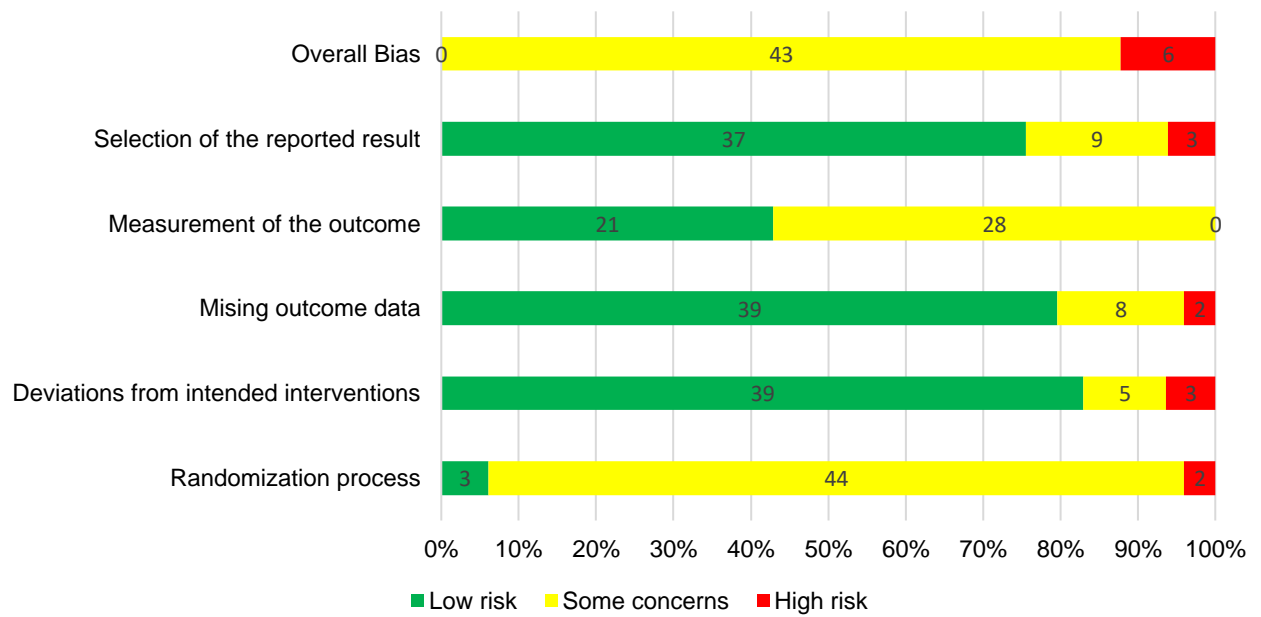

Supplement: Supplementary file 2 — Supplementary Figure S1 (PDF 73 KB) [file 11469_2022_945_MOESM2_ESM.pdf]

### Crossover RCTs: Quality assessment results (in %)

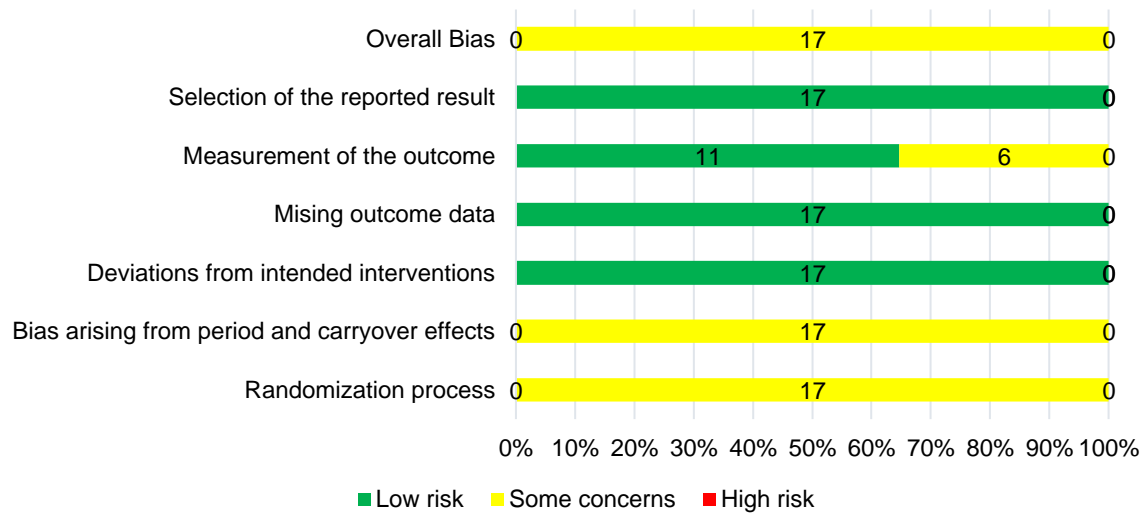

Supplement: Supplementary file 3 — Supplementary Figure S2 (PDF 59 KB) [file 11469_2022_945_MOESM3_ESM.pdf]

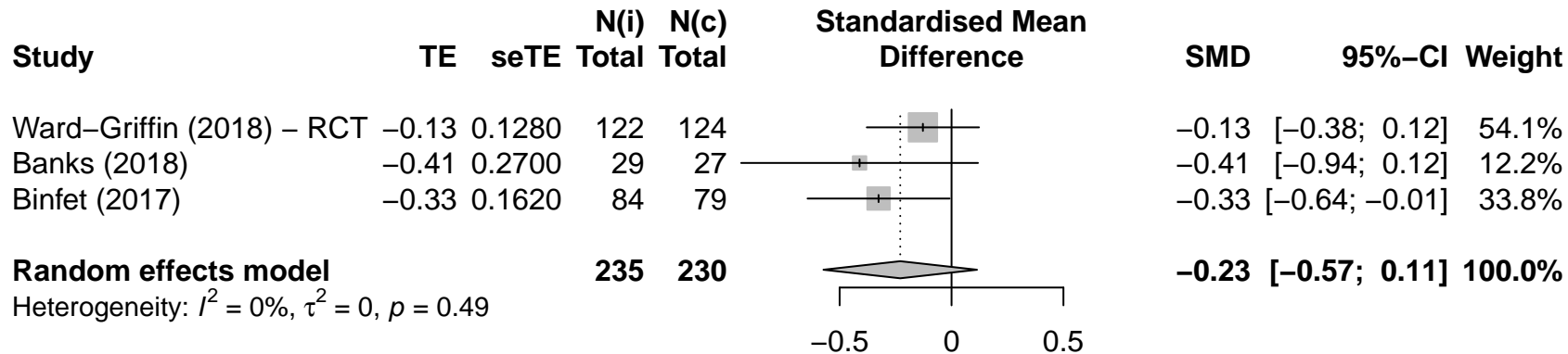

Supplement: Supplementary file 4 — Supplementary Figure S3 (PDF 5 KB) [file 11469_2022_945_MOESM4_ESM.pdf]

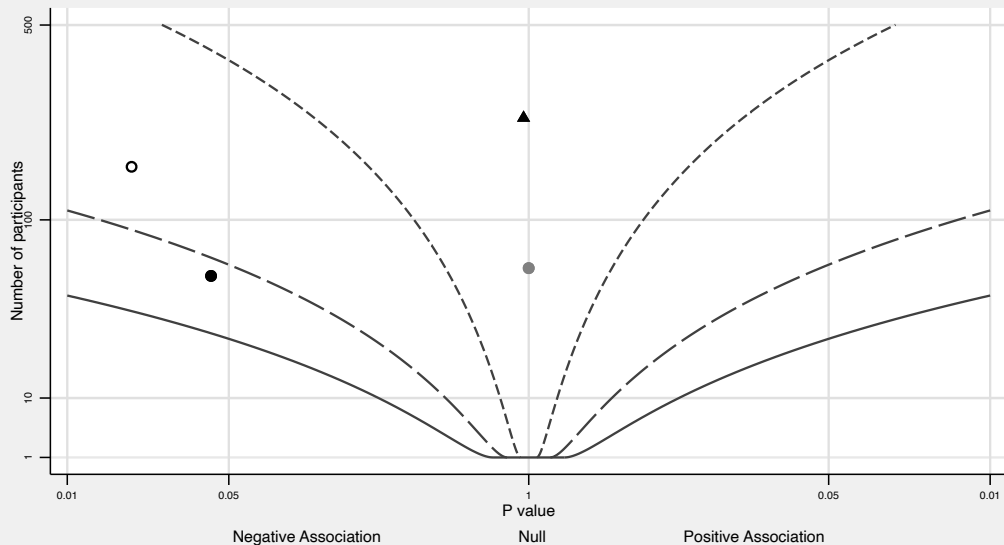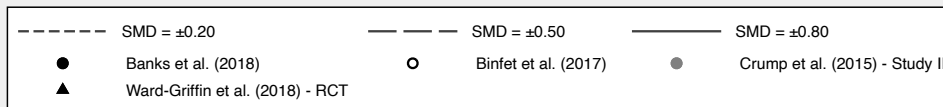

Effect contours drawn using a ratio of group sizes ( $r$ ) of 1.00

Grouped by: Studyauthorsandyear

Supplement: Supplementary file 5 — Supplementary Figure S4 (PDF 39 KB) [file 11469_2022_945_MOESM5_ESM.pdf]

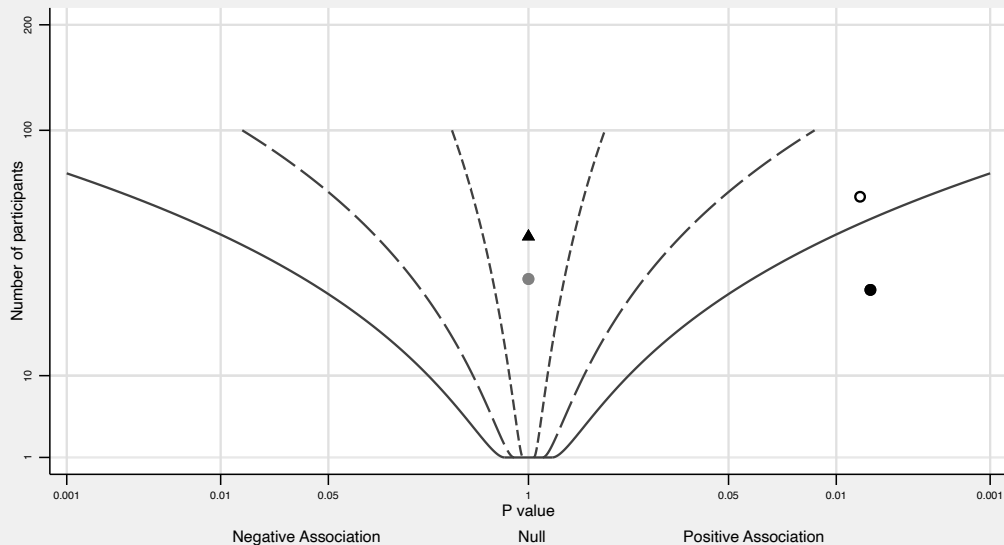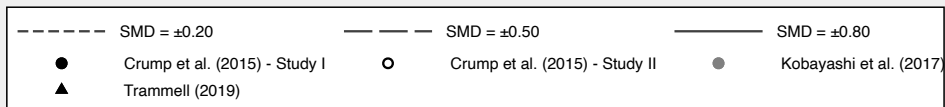

Effect contours drawn using a ratio of group sizes ( $r$ ) of 1.00

Grouped by: Studyauthorsandyear

Supplement: Supplementary file 6 — Supplementary Figure S5 (PDF 38 KB) [file 11469_2022_945_MOESM6_ESM.pdf]

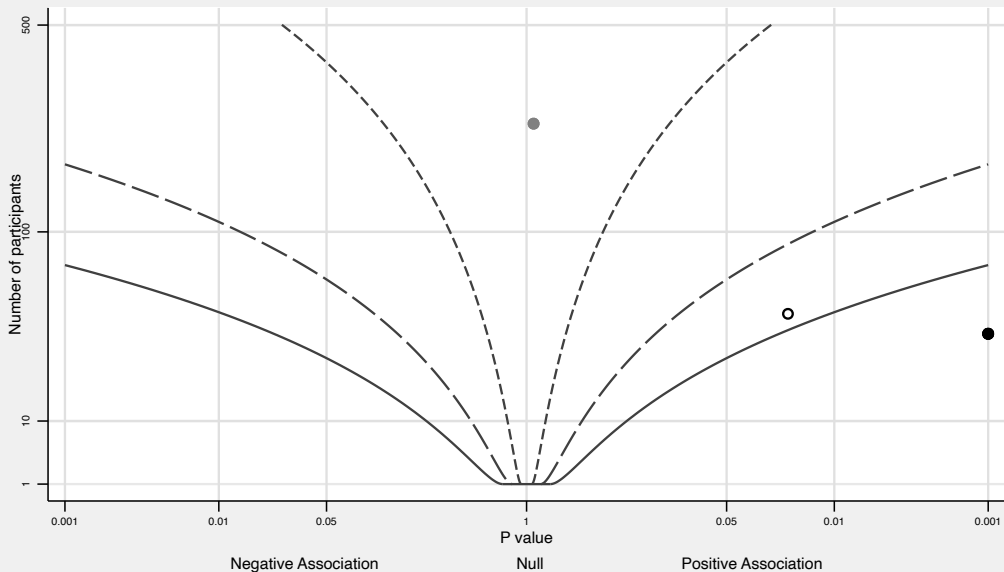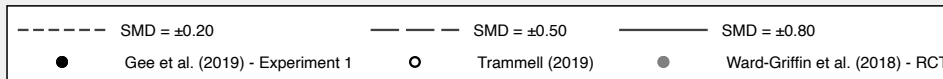

Effect contours drawn using a ratio of group sizes ( $r$ ) of 1.00

Grouped by: Studyauthorsandyear

Supplement: Supplementary file 7 — Supplementary Figure S6 (PDF 39 KB) [file 11469_2022_945_MOESM7_ESM.pdf]

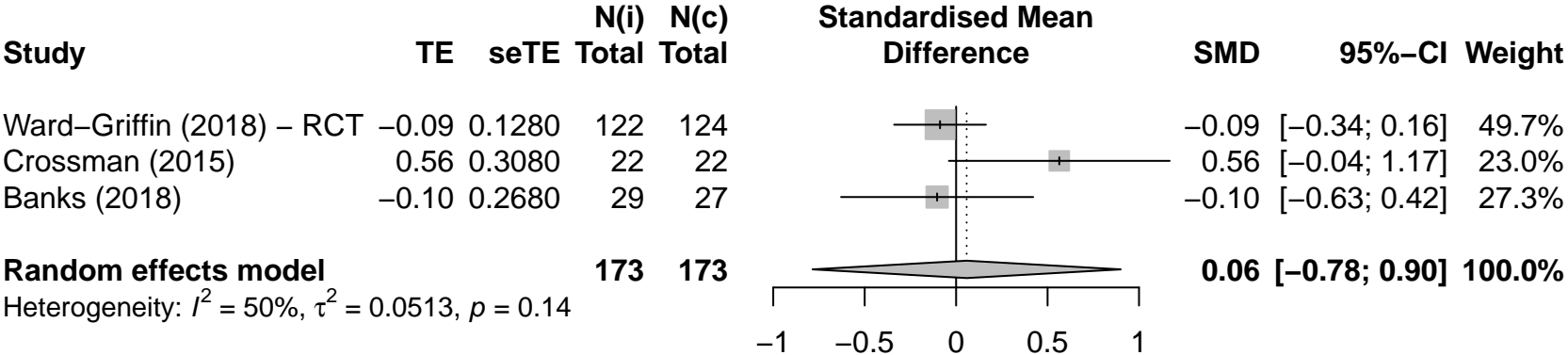

Supplement: Supplementary file 8 — Supplementary Figure S7 (PDF 5 KB) [file 11469_2022_945_MOESM8_ESM.pdf]

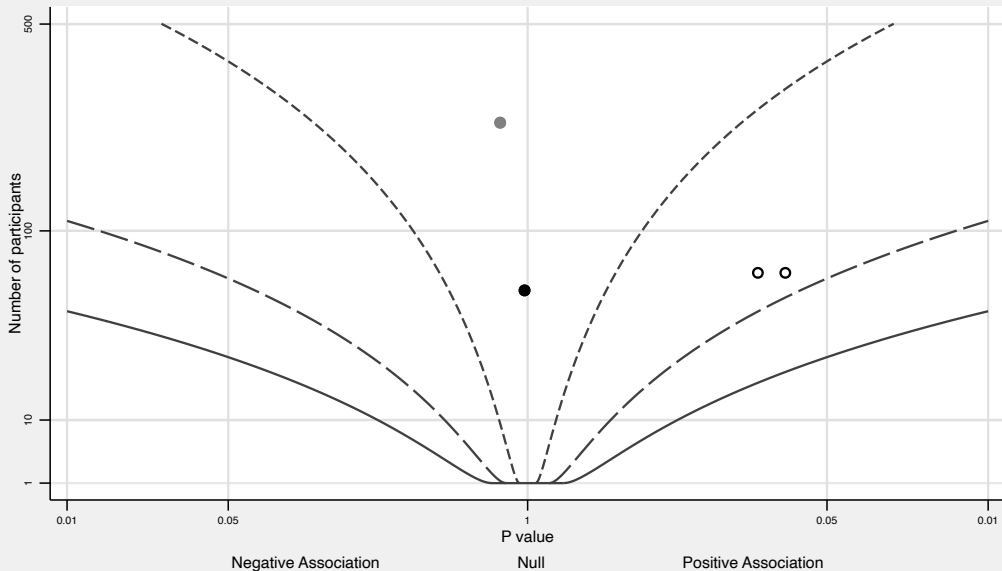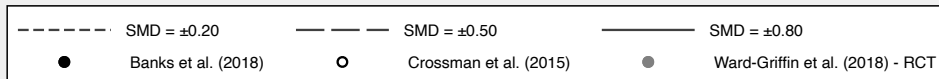

Effect contours drawn using a ratio of group sizes ( $r$ ) of 1.00

Grouped by: Studyauthorsandyear

Supplement: Supplementary file 9 — Supplementary Figure S8 (PDF 39 KB) [file 11469_2022_945_MOESM9_ESM.pdf]

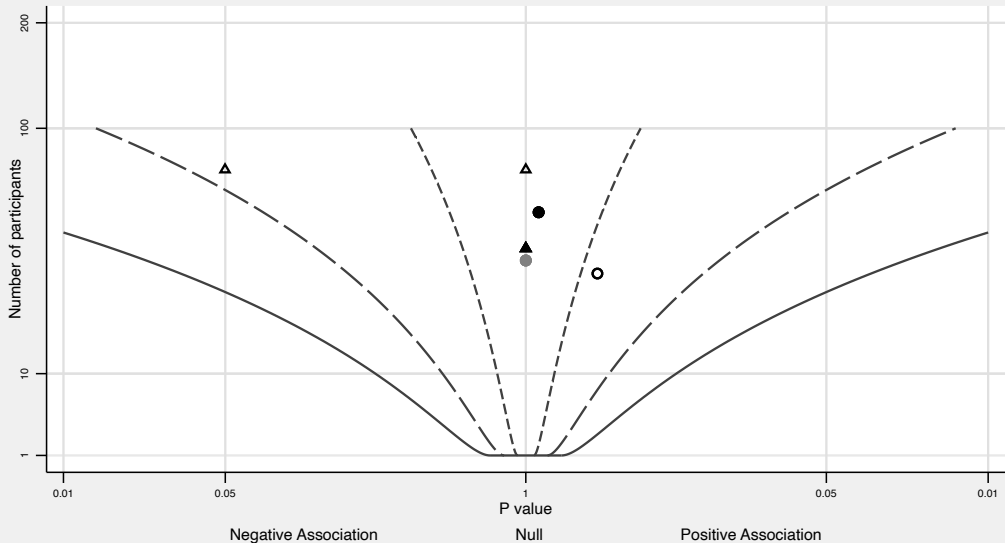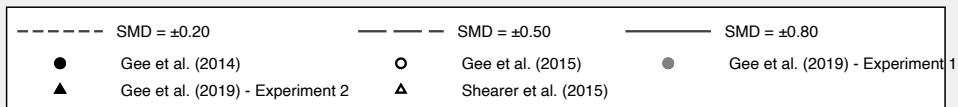

Effect contours drawn using a ratio of group sizes ( $r$ ) of 1.00

Grouped by: Studyauthorsandyear

Supplement: Supplementary file 10 — Supplementary Figure S9 (PDF 38 KB) [file 11469_2022_945_MOESM10_ESM.pdf]

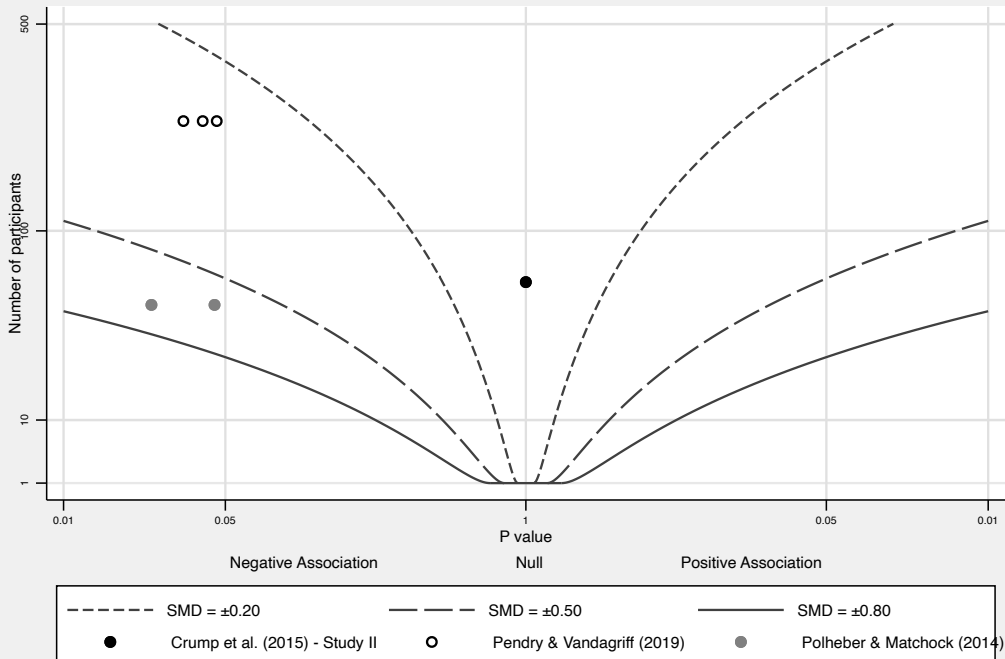

Effect contours drawn using a ratio of group sizes ( $r$ ) of 1.00

Grouped by: Studyauthorsandyear

Supplement: Supplementary file 11 — Supplementary Figure S10 (PDF 39 KB) [file 11469_2022_945_MOESM11_ESM.pdf]

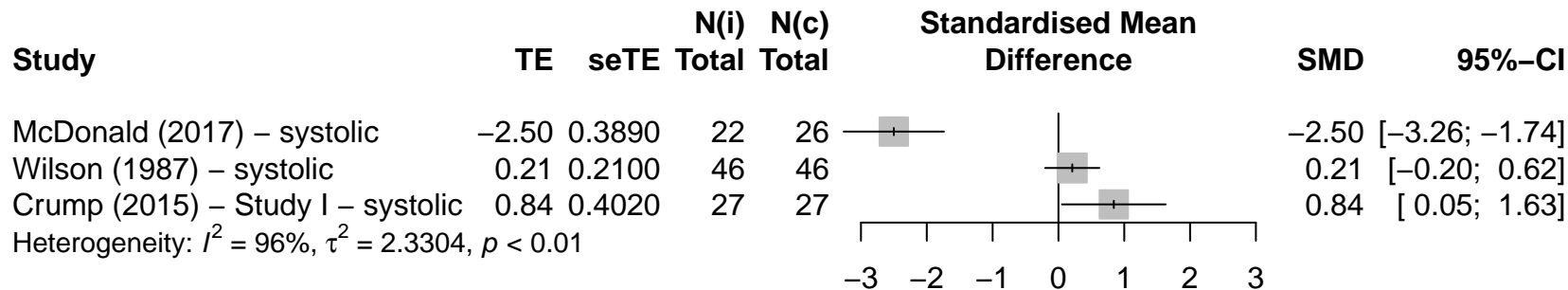

Supplement: Supplementary file 12 — Supplementary Figure S11 (PDF 5 KB) [file 11469_2022_945_MOESM12_ESM.pdf]

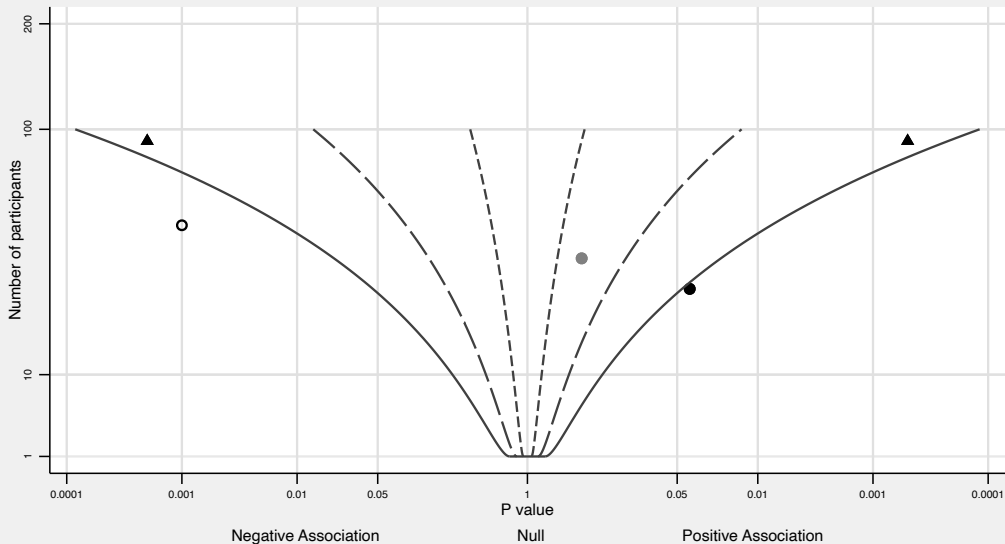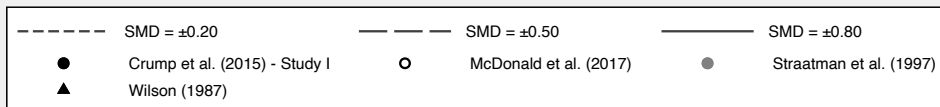

Effect contours drawn using a ratio of group sizes ( $r$ ) of 1.00

Grouped by: Studyauthorsandyear

Supplement: Supplementary file 13 — Supplementary Figure S12 (PDF 38 KB) [file 11469_2022_945_MOESM13_ESM.pdf]

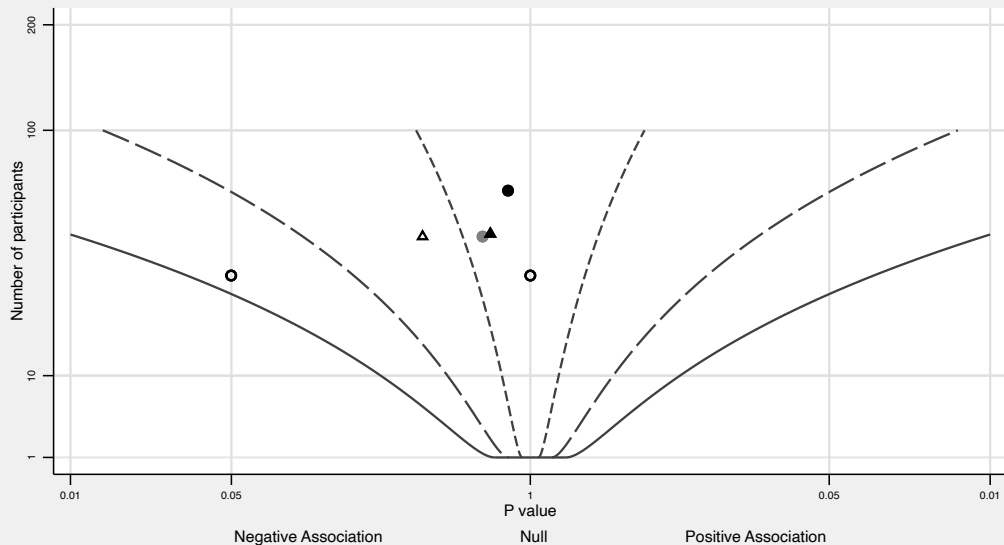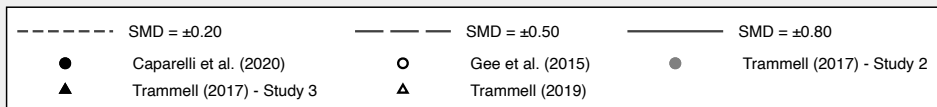

Effect contours drawn using a ratio of group sizes ( $r$ ) of 1.00

Grouped by: Studyauthorsandyear

Supplement: Supplementary file 14 — Supplementary Figure S13 (PDF 39 KB) [file 11469_2022_945_MOESM14_ESM.pdf]

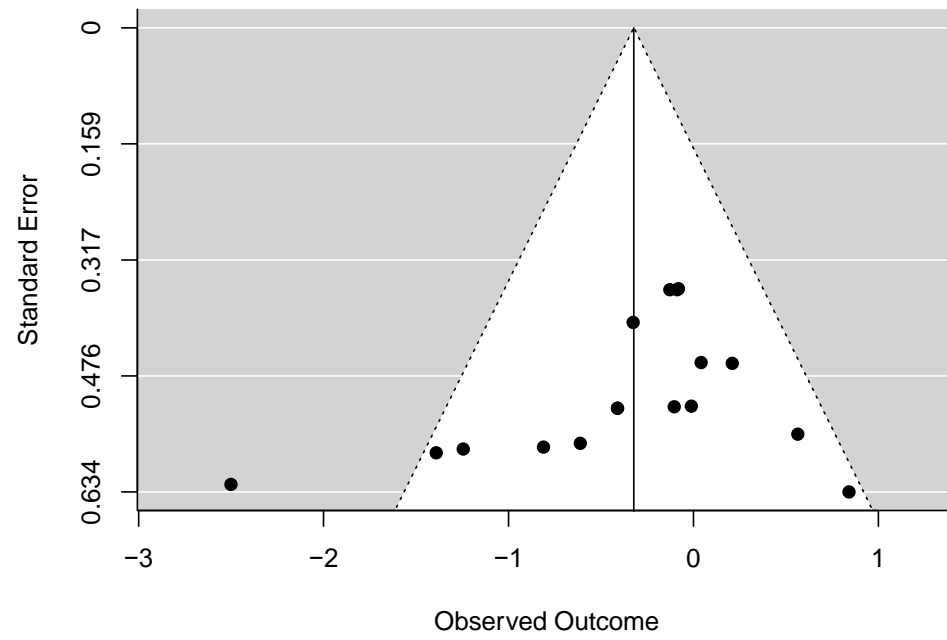

Supplement: Supplementary file 15 — Supplementary Figure S14 (PDF 77 KB) [file 11469_2022_945_MOESM15_ESM.pdf]
