# Supplementary material for: Animal-Assisted Interventions Improve Mental, But Not Cognitive or Physiological Health Outcomes of Higher Education Students: a Systematic Review and Meta-analysis
Source: Int J Ment Health Addict. 2022 Nov 15:1–32. Online ahead of print. doi: 10.1007/s11469-022-00945-4 (PMC9666958; doi:10.1007/s11469-022-00945-4)
Supplement: Supplementary file 17 — Supplementary File3 (PDF 125 KB) [file 11469_2022_945_MOESM17_ESM.pdf]

Appendix S2. List of extracted data items.

- Study title
- Study authors and year
- Study design
    - Study design (RCT or crossover RCT)
    - Type of publication (peer-reviewed journal article or undergraduate/postgraduate/doctoral dissertation)
- Participants
    - Gender
    - Age
    - Number of participants
    - Were sample size calculations conducted?
    - Study location
    - If applicable: pre-existing health condition
    - Were participants pet owners?
    - University degree (subject)
    - University level (Bachelor, Master, PhD)
    - Recruitment process
    - Eligibility criteria for participants
- Intervention
    - Randomization process into intervention and control groups
    - Differences between intervention and control group at baseline
    - Purpose of intervention (physical therapy/stress prevention/anxiety reduction etc.)
    - Was intervention an AAI program integrated in a pre-existing university program?
    - Number of participants in intervention condition
    - Animals in intervention condition
        - Type of animal
        - Breed of animal
        - Training level of animal
        - Number of animals present in intervention condition
    - If applicable: Person administering intervention (researcher, animal handler etc.)
    - If applicable: number of handlers present in intervention condition
    - Animal-to-participant ratio
    - Description of intervention condition
        - Type of activity (presence of animal, active interaction with animal etc.)
        - If applicable: measures used to induce stress among participants (test, interview etc.)
        - Setting of intervention condition
        - Format of intervention condition (groups, individual)
        - Timing of intervention (when in the semester did it take place? Beginning, middle, end, before exams, after exams?)
        - Duration of intervention condition
        - Number of intervention sessions per participant
        - Frequency of individual intervention sessions per participant
        - Duration of individual intervention sessions
- If applicable: Control
    - Number of control conditions
    - Number of participants in control condition(s)
        - Description of control condition(s)

        - Type of activity
        - Setting of control condition
        - Format of control condition (groups, individual)
        - Timing of control (when in the semester did it take place? Beginning, middle, end, before exams, after exams?)
        - Duration of control condition
        - Number of control sessions per participant
        - Frequency of individual control session per participant
        - Duration of individual control session
- Outcomes
    - Quantitative outcomes
        - Outcomes assessed
        - Measures used to assess outcomes
        - Timing of outcome assessments (baseline, immediate at the start of/during intervention, immediately following intervention, long-term)
        - Statistical test used
        - Results from quantitative outcome assessment
        - If available: effect size (Hedge's g) and corresponding SE
        - P-value
        - If applicable: other relevant outcomes from sub-group analyses
    - Qualitative outcomes
        - Outcomes assessed
        - Measures used to assess outcomes
        - Timing of outcome assessments (baseline, immediate at the start of/during intervention, immediately following intervention, long-term)
        - Results from qualitative outcome assessment
