# Supplementary material for: Animal-Assisted Interventions Improve Mental, But Not Cognitive or Physiological Health Outcomes of Higher Education Students: a Systematic Review and Meta-analysis
Source: Int J Ment Health Addict. 2022 Nov 15:1–32. Online ahead of print. doi: 10.1007/s11469-022-00945-4 (PMC9666958; doi:10.1007/s11469-022-00945-4)
Supplement: Supplementary file 18 — Supplementary Table S1 (PDF 53 KB) [file 11469_2022_945_MOESM18_ESM.pdf]

Table SI. Eligibility criteria.

**PICOS** criteria	Eligibility criteria
**P**opulation	• Participants are students of a higher education institution<br>• Participants can be with or without pre-existing health conditions
**I**ntervention	• Using a live animal<br>• Using an animal that is unfamiliar to participants<br>• Animal is the sole intervention tool<br>• Intervention takes place in a higher education setting<br>• Aim of the intervention is to improve any mental, physiological or cognitive outcome of students
**C**ontrol	• Presence of a parallel control group[a]
**O**utcome	• Any mental health outcomes<br>• Any physiological outcomes that are associated with stress[b]<br>• Any cognitive outcomes
**S**tudy design	• Study design must be RCT or crossover RCT with a parallel control group<br>• Study design must include true randomization[c]<br>• Published in a peer-reviewed journal

[a]We defined a parallel control group as a control group that experienced the control condition at the same time as the intervention group experienced the intervention condition. [b]We included physiological outcomes that have been shown to correlate with acute feelings of stress, including heart rate, blood pressure and cortisol levels. [c]We defined randomization as true random allocation to either the intervention and control groups, or in the case of crossover studies, to the order of intervention and control groups.
