# Supplementary material for: Animal-Assisted Interventions Improve Mental, But Not Cognitive or Physiological Health Outcomes of Higher Education Students: a Systematic Review and Meta-analysis
Source: Int J Ment Health Addict. 2022 Nov 15:1–32. Online ahead of print. doi: 10.1007/s11469-022-00945-4 (PMC9666958; doi:10.1007/s11469-022-00945-4)
Supplement: Supplementary file 19 — Supplementary Table S2 (PDF 32 KB) [file 11469_2022_945_MOESM19_ESM.pdf]

Table SII. Overview of search results.

Database searched	Number of retrieved references
Medline (Ovid):	307
Embase (Ovid):	378
ERIC (Ovid)	145
PsycInfo (Ovid):	390
CINAHL (Ebsco):	447
Scopus	835
Web of Science (WoS)	509
OpenGrey (with duplicates)	212
WALTHAM Scientific Publications	31
	95
**Number of references before deduplication:**	**3759**
**Number of references after deduplication:**	**2431**
