# Supplementary material for: Animal-Assisted Interventions Improve Mental, But Not Cognitive or Physiological Health Outcomes of Higher Education Students: a Systematic Review and Meta-analysis
Source: Int J Ment Health Addict. 2022 Nov 15:1–32. Online ahead of print. doi: 10.1007/s11469-022-00945-4 (PMC9666958; doi:10.1007/s11469-022-00945-4)
Supplement: Supplementary file 20 — Supplementary Table S3 (PDF 75 KB) [file 11469_2022_945_MOESM20_ESM.pdf]

Table SIII. Coded table for acute self-perceived stress (n=7).

Study authors and year	RoB 2.0 score	Hedges‘ g and SE available?	Animal used in intervention condition		Type of intervention condition		Type of control condition			
			Dog	Other	Active intervention	Passive intervention	No treatment	Animal	Human	Other
Trammell (2019)	Some concerns	Yes	Dog			Passive intervention	No treatment			
Trammell (2017) - Study 2	Some concerns	Yes	Dog		Active intervention			Animal		
Trammell (2017) - Study 3	Some concerns	Yes	Dog		Active intervention			Animal		
Gebhart et al. (2018)	Some concerns	No	Dog		Active intervention		No treatment			Other
Barker et al. (2016)	Some concerns	No	Dog		Active intervention		No treatment			
Crump et al. (2015) - Study I	Some concerns	Yes	Dog		Active intervention		No treatment			
Crump et al. (2015) - Study II	Some concerns	Yes	Dog		Active intervention		No treatment			
