# Supplementary material for: Animal-Assisted Interventions Improve Mental, But Not Cognitive or Physiological Health Outcomes of Higher Education Students: a Systematic Review and Meta-analysis
Source: Int J Ment Health Addict. 2022 Nov 15:1–32. Online ahead of print. doi: 10.1007/s11469-022-00945-4 (PMC9666958; doi:10.1007/s11469-022-00945-4)
Supplement: Supplementary file 23 — Supplementary Table S6 (PDF 139 KB) [file 11469_2022_945_MOESM23_ESM.pdf]

**Table SVI: Coded table for acute anxiety (n=11).**

| **Study authors and year** | **RoB 2.0 score** | **Hedges‘ g and SE available?** | **Animal used in intervention condition** | | **Type of intervention condition** | | **Type of control condition** | | | |
|---|---|---|---|---|---|---|---|---|---|---|
| | | | Dog | Other | Active intervention | Passive intervention | No treatment | Animal | Human | Other |
| **Banks et al. (2018)** | **Some concerns** | **Yes** | **Dog** | | **Active intervention** | | **No treatment** | | | |
| **Crossman et al. (2015)** | **Some concerns** | **Yes** | **Dog** | | **Active intervention** | | **No treatment** | Animal | | |
| Gebhart et al. (2018) | Some concerns | No | Dog | | Active intervention | | No treatment | | | Other |
| Gee et al. (2019) - Experiment 2 | Some concerns | Yes | | Fish | Active intervention | | No treatment | Animal | | |
| Grajfoner et al. (2017) | Some concerns | Yes | Dog | | Active intervention | | | Animal | Human | |
| Hall (2018) | High risk | Yes | Dog | | Active intervention | | No treatment | | | |
| Pendry et al. (2018) | High risk | Yes | Dogs, cats | | Active intervention | | No treatment | Animal | | |
| Polheber & Matchock (2014) | Some concerns | No | Dog | | | Passive intervention | No treatment | | Human | |
| **Shearer et al. (2015)** | **Some concerns** | **Yes** | **Dog** | | **Active intervention** | | **No treatment** | | | Other |

| | | | | | | | | | |
|---|---|---|---|---|---|---|---|---|---|
| Stewart & Strickland (2013) | Some concerns | Yes | Dog | | | Passive intervention | No treatment | | |
| **Wilson (1987)** | **Some concerns** | **Yes** | **Dog** | | **Active intervention** | | **No treatment** | | Other |

Studies highlighted in bold were included in the meta-analyses.
