# Supplementary material for: Animal-Assisted Interventions Improve Mental, But Not Cognitive or Physiological Health Outcomes of Higher Education Students: a Systematic Review and Meta-analysis
Source: Int J Ment Health Addict. 2022 Nov 15:1–32. Online ahead of print. doi: 10.1007/s11469-022-00945-4 (PMC9666958; doi:10.1007/s11469-022-00945-4)
Supplement: Supplementary file 24 — Supplementary Table S7 (PDF 73 KB) [file 11469_2022_945_MOESM24_ESM.pdf]

Table SVII: Coded table for arousal (n=4).

Study authors and year	RoB 2.0 score	Hedges' g and SE available?	Animal used in intervention condition		Type of intervention condition		Type of control condition			
			Dog	Other	Active intervention	Passive intervention	No treatment	Animal	Human	Other
Crump et al. (2015) - Study I	Some concerns	Yes	Dog		Active intervention		No treatment			
Crump et al. (2015) - Study II	Some concerns	Yes	Dog		Active intervention		No treatment			
Kobayashi et al. (2017)	Some concerns	Yes		Cat	Active intervention		No treatment			
Trammell (2019)	Some concerns	Yes	Dog			Passive intervention	No treatment			
