# Supplementary material for: Animal-Assisted Interventions Improve Mental, But Not Cognitive or Physiological Health Outcomes of Higher Education Students: a Systematic Review and Meta-analysis
Source: Int J Ment Health Addict. 2022 Nov 15:1–32. Online ahead of print. doi: 10.1007/s11469-022-00945-4 (PMC9666958; doi:10.1007/s11469-022-00945-4)
Supplement: Supplementary file 26 — Supplementary Table S9 (PDF 77 KB) [file 11469_2022_945_MOESM26_ESM.pdf]

Table SIX: Coded table for positive affect (n=3).

Study authors and year	RoB 2.0 score	Hedges' g and SE available?	Animal		Type of intervention activity		Type of control condition			
			Dog	Other	Active intervention	Passive intervention	No treatment	Animal	Human	Other
**Banks et al. (2018)**	**Some concerns**	**Yes**	**Dog**		**Active intervention**		**No treatment**			
**Crossman et al. (2015)**	**Some concerns**	**Yes**	**Dog**		**Active intervention**		**No treatment**	Animal		
**Ward-Griffin et al. (2018) - RCT**	**Some concerns**	**Yes**	**Dog**		**Active intervention**		**No treatment**			

Studies highlighted in bold were included in the meta-analyses.
