# Supplementary material for: Animal-Assisted Interventions Improve Mental, But Not Cognitive or Physiological Health Outcomes of Higher Education Students: a Systematic Review and Meta-analysis
Source: Int J Ment Health Addict. 2022 Nov 15:1–32. Online ahead of print. doi: 10.1007/s11469-022-00945-4 (PMC9666958; doi:10.1007/s11469-022-00945-4)
Supplement: Supplementary file 27 — Supplementary Table S10 (PDF 78 KB) [file 11469_2022_945_MOESM27_ESM.pdf]

Table SX: Coded table for blood pressure (n=4).

Study authors and year	RoB 2.0 score	Hedges' g and SE available?	Animal used in intervention condition		Type of intervention condition		Type of control condition			
			Dog	Other	Active intervention	Passive intervention	No treatment	Animal	Human	Other
**Crump et al. (2015) - Study I**	**Some concerns**	**Yes**	**Dog**		**Active intervention**		**No treatment**			
**McDonald et al. (2017)**	**Some concerns**	**Yes**	**Dog**		**Active intervention**		**No treatment**			
Straatman et al. (1997)	Some concerns	No	Dog			Passive intervention	No treatment			
**Wilson (1987)**	**Some concerns**	**Yes**	**Dog**		**Active intervention**		**No treatment**			Other

Studies highlighted in bold were included in the meta-analyses.
