# Supplementary material for: Animal-Assisted Interventions Improve Mental, But Not Cognitive or Physiological Health Outcomes of Higher Education Students: a Systematic Review and Meta-analysis
Source: Int J Ment Health Addict. 2022 Nov 15:1–32. Online ahead of print. doi: 10.1007/s11469-022-00945-4 (PMC9666958; doi:10.1007/s11469-022-00945-4)
Supplement: Supplementary file 28 — Supplementary Table S11 (PDF 123 KB) [file 11469_2022_945_MOESM28_ESM.pdf]

## Table SXI: Coded table for heart rate (n=8).

| Study authors and year | RoB 2.0 score | Hedges' g and SE available? | Animal used in intervention condition | | Type of intervention condition | | Type of control condition | | | |
|---|---|---|---|---|---|---|---|---|---|---|
| | | | Dog | Other | Active intervention | Passive intervention | No treatment | Animal | Human | Other |
| Crump et al. (2015) - Study I | Some concerns | No | Dog | | Active intervention | | No treatment | | | |
| Gee et al. (2014) | Some concerns | No | Dog | | | Passive intervention | | Animal | Human | |
| Gee et al. (2015) | Some concerns | No | Dog | | | Passive intervention | No treatment | Animal | Human | |
| Gee et al. (2019) - Experiment 1 | Some concerns | Yes | | Fish | Active intervention | | No treatment | Animal | | |
| Gee et al. (2019) - Experiment 2 | Some concerns | Yes | | Fish | Active intervention | | No treatment | Animal | | |
| Polheber & Matchock (2014) | Some concerns | No | Dog | | | Passive intervention | No treatment | | Human | |
| Straatman et al. (1997) | Some concerns | No | Dog | | | Passive intervention | No treatment | | | |
| Wilson (1987) | Some concerns | Yes | Dog | | Active intervention | | No treatment | | | Other |
