# Supplementary material for: Animal-Assisted Interventions Improve Mental, But Not Cognitive or Physiological Health Outcomes of Higher Education Students: a Systematic Review and Meta-analysis
Source: Int J Ment Health Addict. 2022 Nov 15:1–32. Online ahead of print. doi: 10.1007/s11469-022-00945-4 (PMC9666958; doi:10.1007/s11469-022-00945-4)
Supplement: Supplementary file 29 — Supplementary Table S12 (PDF 74 KB) [file 11469_2022_945_MOESM29_ESM.pdf]

Table SXII: Coded table for heart rate variability (n=5).

Study authors and year	RoB 2.0 score	Hedges' g and SE available?	Animal used in intervention condition		Type of intervention condition		Type of control condition			
			Dog	Other	Active intervention	Passive intervention	No treatment	Animal	Human	Other
Gee et al. (2014)	Some concerns	No	Dog			Passive intervention		Animal	Human	
Gee et al. (2015)	Some concerns	No	Dog			Passive intervention	No treatment	Animal	Human	
Gee et al. (2019) - Experiment 1	Some concerns	Yes		Fish	Active intervention		No treatment	Animal		
Gee et al. (2019) - Experiment 2	Some concerns	Yes		Fish	Active intervention		No treatment	Animal		
Shearer et al. (2015)	Some concerns	Yes	Dog		Active intervention		No treatment			Other
