# Supplementary material for: Animal-Assisted Interventions Improve Mental, But Not Cognitive or Physiological Health Outcomes of Higher Education Students: a Systematic Review and Meta-analysis
Source: Int J Ment Health Addict. 2022 Nov 15:1–32. Online ahead of print. doi: 10.1007/s11469-022-00945-4 (PMC9666958; doi:10.1007/s11469-022-00945-4)
Supplement: Supplementary file 30 — Supplementary Table S13 (PDF 72 KB) [file 11469_2022_945_MOESM30_ESM.pdf]

Table SXIII: Coded table for salivary cortisol (n=3).

**Study authors and year**	**RoB 2.0 score**	**Hedges' g and SE available?**	**Animal used in intervention condition**		**Type of intervention condition**		**Type of control condition**			
			Dog	Other	Active intervention	Passive intervention	No treatment	Animal	Human	Other
Crump et al. (2015) - Study II	Some concerns	No	Dog		Active intervention		No treatment			
Pendry & Vandagriff (2019)	Some concerns	Yes	Dogs, cats		Active intervention		No treatment	Animal		
Polheber & Matchock (2014)	Some concerns	No	Dog			Passive intervention	No treatment		Human	
