# Supplementary material for: Animal-Assisted Interventions Improve Mental, But Not Cognitive or Physiological Health Outcomes of Higher Education Students: a Systematic Review and Meta-analysis
Source: Int J Ment Health Addict. 2022 Nov 15:1–32. Online ahead of print. doi: 10.1007/s11469-022-00945-4 (PMC9666958; doi:10.1007/s11469-022-00945-4)
Supplement: Supplementary file 31 — Supplementary Table S14 (PDF 76 KB) [file 11469_2022_945_MOESM31_ESM.pdf]

Table SXIV: Coded table for performance on a memory task (n=5).

**Study authors and year**	**RoB 2.0 score**	**Hedges‘ g and SE available?**	**Animal used in intervention condition**		**Type of intervention condition**		**Type of control condition**			
			Dog	Other	Active intervention	Passive intervention	No treatment	Animal	Human	Other
Capparelli et al. (2020)	Some concerns	Yes	Dog			Passive intervention (dog present during task)	No treatment			
Gee et al. (2015)	Some concerns	No	Dog			Passive intervention (dog present during task)	No treatment		Human	
Trammell (2017) - Study 2	Some concerns	Yes	Dog		Active intervention (dog present immediately before but not during task)			Animal		
Trammell (2017) - Study 3	Some concerns	Yes	Dog		Active intervention (dog present after preparing for but not during task)			Animal		
Trammell (2019)	Some concerns	Yes	Dog			Passive intervention (dog present while preparing for but not during task)	No treatment			
