# Supplementary material for: Animal-Assisted Interventions Improve Mental, But Not Cognitive or Physiological Health Outcomes of Higher Education Students: a Systematic Review and Meta-analysis
Source: Int J Ment Health Addict. 2022 Nov 15:1–32. Online ahead of print. doi: 10.1007/s11469-022-00945-4 (PMC9666958; doi:10.1007/s11469-022-00945-4)
Supplement: Supplementary file 32 — Supplementary Table S15 (PDF 73 KB) [file 11469_2022_945_MOESM32_ESM.pdf]

Table SXV. Calculations for meta-analysis.

Reference	Outcomes included in meta-analysis	Input used („esc“ function used)	Hedges‘ g and SE
Banks et al. (2018)	Chronic self-perceived stress<br><br>Acute anxiety<br><br>Positive affect<br><br>Negative affect	means and sds (esc_mean_sd)	Chronic self-perceived stress: g= -0.411, SE= 0.27<br><br>Acute anxiety: g= -0.52, SE= 0.272<br><br>Positive affect: g= -0.104, SE= 0.268<br><br>Negative affect: g= -0.011, SE= 0.267
Binfet et al. (2017)	Chronic self-perceived stress	means and sds (esc_mean_sd)	g= -0.326, SE= 0.162
Crossman et al. (2015)	Acute anxiety<br><br>Positive affect<br><br>Negative affect	means and sds (esc_mean_sd)	Acute anxiety: g= -1.245, SE= 0.331<br><br>Negative affect: g= -1.391, SE=0.337<br><br>Positive affect: g= 0.564, SE= 0.308
Crump et al. (2015) - Study I	Systolic blood pressure	f-test (esc_f)	Systolic blood pressure: g= 0.814, SE= 0.402
McDonald et al. (2017)	Systolic blood pressure	means and sds (esc_mean_sd)	g= -2.501, SE= 0.389
Shearer et al. (2015)	Acute anxiety<br><br>Negative affect	means and sds at time point 4 (esc_mean_sd)	Acute anxiety: g= -0.811, SE= 0.328<br><br>Negative affect: g= -0.612, SE= 0.322

Ward-Griffin et al. (2018) - RCT	Positive affect<br><br>Negative affect<br><br>Chronic self-perceived stress	means and sds (esc_mean_sd)	Positive affect: g= -0.088, SE= 0.128<br><br>Negative affect: g= -0.081, SE= 0.127<br><br>Chronic self-perceived stress: g= -0.128, SE= 0.128
Wilson (1987)	Systolic blood pressure<br><br>Acute anxiety	means and sds (esc_mean_sd) (intervention vs. reading quietly)	Systolic blood pressure: g= 0.21, SE= 0.21<br><br>Acute anxiety: g= 0.0413, SE= 0.209
