# Supplementary material for: Animal-Assisted Interventions Improve Mental, But Not Cognitive or Physiological Health Outcomes of Higher Education Students: a Systematic Review and Meta-analysis
Source: Int J Ment Health Addict. 2022 Nov 15:1–32. Online ahead of print. doi: 10.1007/s11469-022-00945-4 (PMC9666958; doi:10.1007/s11469-022-00945-4)
Supplement: Supplementary file 33 — Supplementary Table S16 (PDF 99 KB) [file 11469_2022_945_MOESM33_ESM.pdf]

Table SXVI. Supplemental data extraction table.

Reference	Gender	Location	Type of intervention condition	Type of control condition	Stressor?[a]	Format[b]	Frequency of sessions[c]	Duration of sessions (mins)
Banks et al. (2018)	Mostly female	USA	Active	No treatment	Before exams	Group	1	10
Barker et al. (2016)	Mostly female	USA	Active	No treatment	Before exams	Group	1	15
Barker et al. (2017)	Mostly female	USA	Active	No treatment	Before exams	Group	1	15
Binfet et al. (2017)	Mostly female	Canada	Active	No treatment	No	Group	1	20
Caparelli et al. (2020)	Mostly female	USA	Passive	No treatment	No	Individual	1	6
Charnetski et al. (2004)	n/s	USA	Active	(1) animal, (2) no treatment	No	Individual	1	18
Crossman et al. (2015)	Half male, half female	USA	Active	(1) animal, (2) no treatment	No	Individual	1	7
Crump et al. (2015) - Study I	All female	USA	Active	No treatment	Before exams	Group	1	15
Crump et al. (2015) - Study II	All female	USA	Active	No treatment	Before exams	Group	1	30
Fiocco & Hunse (2017)	Mostly female	Canada	Active	No treatment	Yes	Individual	1	10
Gebhart et al. (2019)	Mostly female	Austria	Active	(1 and 2) other, (3) no treatment	Before exams	Group	>1	45
Gee et al. (2014)	Mostly female	USA	Passive	(1) human, (2) animal	Yes	Individual	1	3
Gee et al. (2015)	Mostly female	USA	Passive	(1 and 2) human, (3) no treatment	Yes	Individual	1	3
Gee et al. (2019) - Experiment 1	Mostly female	USA	Active	(1) animal, (2) no treatment	Yes	Individual	1	5

Gee et al. (2019) - Experiment 2	Mostly female	USA	Active	(1) animal, (2) no treatment	Yes	Individual	1	5
Gonzalez-Ramirez et al. (2016)	Half male, half female	Mexico	Active	No treatment	Yes	Group	n/s	n/s
Grajfoner et al. (2017)	Mostly female	Scotland	Active	(1) animal, (2) human	No	Group	1	20
Hall (2018)	Mostly female	USA	Active	No treatment	No	Group or individual	n/s	n/s
Hunt & Chizkov (2014)	Mostly female	USA	Passive	(1 and 2) no treatment	Yes	Individual	>1	20
Kobayashi et al. (2017)	Mostly female	Japan	Active	Animal	No	Individual	1	1
McDonald et al. (2017)	n/s	USA	Active	No treatment	Before exams	Group	1	15
Pendry & Vandagriff (2019)	Mostly female	USA	Active	(1) animal, (2 and 3) no treatment	Before exams	Dogs: group, cats: individual	1	10
Pendry et al. (2018)	Mostly female	USA	Active	(1) animal, (2) no treatment	Before exams	Dogs: group, cats: individual	1	10
Pendry et al. (2019)	Mostly female	USA	Active	(1 and 2) other	No	Group	>1	60
Pendry et al. (2019, Clinical depression)	Mostly female	USA	Active	(1) animal, (2) no treatment	Before exams	Dogs: group, cats: individual	1	10
Pendry et al. (2020)	Mostly female	USA	Active	(1 and 2) other	No	Group	>1	60
Polheber & Matchock (2014)	Half male, half female	USA	Passive	(1) human, (2) no treatment	Yes	Individual	1	53
Shearer et al. (2015)	Half male, half female	USA	Active	(1) other, (2) no treatment	No	Group	>1	60

Stewart & Strickland (2013)	Mostly female	USA	Passive	No treatment	Yes	Individual	1	14
Straatman et al. (1997)	All male	Netherlands	Passive	No treatment	Yes	Individual	1	36
Trammell (2017) - Study 2	n/s	USA	Active	Animal	Before exams	Group	1	15
Trammell (2017) - Study 3	n/s	USA	Active	Animal	Before exams	Group	1	15
Trammell (2019)	Mostly female	USA	Passive	No treatment	Yes	Group	>1	n/s
Ward-Griffin et al. (2018)	Mostly female	Canada	Active	No treatment	No	Group	1	30
Wilson (1987)	Mostly female	USA	Active	(1) other, (2) no treatment	No	Individual	1	10

n/s: not specified. [a]Whether the intervention included a stressor (yes), no stressor (no) or took place shortly before exams. [b]Whether the intervention took place in a group or individual format. [c]Frequency sessions: whether sessions took place once (1) or more than once (>1).
