# Supplementary material for: Animal-Assisted Interventions Improve Mental, But Not Cognitive or Physiological Health Outcomes of Higher Education Students: a Systematic Review and Meta-analysis
Source: Int J Ment Health Addict. 2022 Nov 15:1–32. Online ahead of print. doi: 10.1007/s11469-022-00945-4 (PMC9666958; doi:10.1007/s11469-022-00945-4)
Supplement: Supplementary file 34 — Supplementary Table S17 (PDF 92 KB) [file 11469_2022_945_MOESM34_ESM.pdf]

Table SXVII. Quality assessment results for RCTs at the individual outcome level (n(outcomes)=49).

Reference	Outcome	Effect of adhering to intervention?	Randomization process	Deviations from intended interventions	Mising outcome data	Measurement of the outcome	Selection of the reported result	Overall Bias
**Banks et al. (2018)**	Chronic self-perceived stress	Effect of assignment to intervention	Some concerns	Low risk	Low risk	Some concerns	Low risk	Some concerns
Banks et al. (2018)	Positive and negative affect	Effect of assignment to intervention	Some concerns	Low risk	Low risk	Some concerns	Low risk	Some concerns
Banks et al. (2018)	Acute anxiety	Effect of assignment to intervention	Some concerns	Low risk	Low risk	Some concerns	Low risk	Some concerns
**Binfet et al. (2017)**	Chronic self-perceived stress	Effect of assignment to intervention	Some concerns	Low risk	Some concerns	Some concerns	Low risk	Some concerns
**Caparelli et al. (2020)**	Memory test	Effect of assignment to intervention	Some concerns	Some concerns	Low risk	Low risk	Some concerns	Some concerns
**Crossman et al. (2015)**	Acute anxiety	Effect of assignment to intervention	Some concerns	Low risk	Low risk	Some concerns	Low risk	Some concerns
Crossman et al. (2015)	Positive and negative affect	Effect of assignment to intervention	Some concerns	Low risk	Low risk	Some concerns	Low risk	Some concerns
**Crump et al. (2015) - Study II**	Acute self-perceived stress	Effect of assignment to intervention	Some concerns	Low risk	Low risk	Some concerns	Low risk	Some concerns
Crump et al. (2015) - Study II	Arousal	Effect of assignment to intervention	Some concerns	Low risk	Low risk	Some concerns	Low risk	Some concerns
Crump et al. (2015) - Study II	Chronic self-perceived stress	Effect of assignment to intervention	Some concerns	Low risk	Low risk	Some concerns	Low risk	Some concerns
Crump et al. (2015) - Study II	Salivary cortisol	Effect of assignment to intervention	Some concerns	Low risk	Low risk	Low risk	Low risk	Some concerns
**Fiocco & Hunse (2017)**	Positive and negative affect	Effect of assignment to intervention	Some concerns	Low risk	Low risk	Some concerns	Low risk	Some concerns
**Gebhart et al. (2018)**	Acute anxiety	Effect of assignment to intervention	Low risk	Low risk	Some concerns	Some concerns	Low risk	Some concerns
Gebhart et al. (2018)	Acute self-perceived stress	Effect of assignment to intervention	Low risk	Low risk	Some concerns	Some concerns	Low risk	Some concerns
Gebhart et al. (2018)	Salivary cortisol	Effect of assignment to intervention	Low risk	Low risk	Some concerns	Low risk	Low risk	Some concerns
**Gee et al. (2019) - Experiment 2**	Acute anxiety	Effect of assignment to intervention	Some concerns	Low risk	Low risk	Some concerns	Low risk	Some concerns
Gee et al. (2019) - Experiment 2	HR	Effect of assignment to intervention	Some concerns	Low risk	Low risk	Low risk	Low risk	Some concerns
Gee et al. (2019) - Experiment 2	HRV	Effect of assignment to intervention	Some concerns	Low risk	Low risk	Low risk	Low risk	Some concerns

**Grajfoner et al. (2017)**	Acute anxiety	Effect of assignment to intervention	Some concerns	Low risk	Low risk	Some concerns	Low risk	Some concerns
**Hall (2018)**	Acute anxiety	Effect of assignment to intervention	Some concerns	High risk	Some concerns	Some concerns	Low risk	High risk
**Hunt & Chizkov (2015)**	Acute anxiety	Effect of assignment to intervention	Some concerns	Low risk	Low risk	Some concerns	High risk	High risk
Hunt & Chizkov (2015)	Positive and negative affect	Effect of assignment to intervention	Some concerns	Low risk	Low risk	Some concerns	High risk	High risk
**McDonald et al. (2017)**	BP	Effect of assignment to intervention	Some concerns	Some concerns	Low risk	Low risk	Some concerns	Some concerns
**Pendry & Vandagriff (2019)**	Salivary cortisol	Effect of assignment to intervention	Some concerns	Low risk	Low risk	Low risk	Low risk	Some concerns
**Pendry et al. (2018)**	Acute anxiety	Effect of assignment to intervention	High risk	High risk	High risk	Low risk	Low risk	High risk
Pendry et al. (2018)	Acute depression	Effect of assignment to intervention	High risk	High risk	High risk	Low risk	Low risk	High risk
**Pendry et al. (2019)**	Acute anxiety	Effect of assignment to intervention	Some concerns	Low risk	Low risk	Some concerns	Low risk	Some concerns
Pendry et al. (2019)	Acute depression	Effect of assignment to intervention	Some concerns	Low risk	Low risk	Some concerns	Low risk	Some concerns
**Polheber & Matchock (2014)**	HR	Effect of assignment to intervention	Some concerns	Low risk	Low risk	Low risk	Low risk	Some concerns
Polheber & Matchock (2014)	Salivary cortisol	Effect of assignment to intervention	Some concerns	Low risk	Low risk	Low risk	Low risk	Some concerns
Polheber & Matchock (2014)	Acute anxiety	Effect of assignment to intervention	Some concerns	Low risk	Low risk	Some concerns	Low risk	Some concerns
**Shearer et al. (2015)**	Acute anxiety	Effect of assignment to intervention	Some concerns	Some concerns	Some concerns	Some concerns	Some concerns	Some concerns
Shearer et al. (2015)	Negative affect	Effect of assignment to intervention	Some concerns	Some concerns	Some concerns	Some concerns	Some concerns	Some concerns
Shearer et al. (2015)	HRV	Effect of assignment to intervention	Some concerns	Some concerns	Low risk	Low risk	Low risk	Some concerns
**Stewart & Strickland (2013)**	Acute anxiety	Effect of assignment to intervention	Some concerns	Low risk	Low risk	Low risk	Some concerns	Some concerns
**Straatman et al. (1997)**	HR	Effect of assignment to intervention	Some concerns	Low risk	Low risk	Low risk	Low risk	Some concerns
Straatman et al. (1997)	BP	Effect of assignment to intervention	Some concerns	Low risk	Low risk	Low risk	Low risk	Some concerns
Straatman et al. (1997)	Acute anxiety	Effect of assignment to intervention	Some concerns	Low risk	Low risk	Some concerns	High risk	High risk
**Trammell (2017) - Study 2**	Acute self-perceived stress	Effect of assignment to intervention	Some concerns	Low risk	Low risk	Some concerns	Low risk	Some concerns
Trammell (2017) - Study 2	Memory test	Effect of assignment to intervention	Some concerns	Low risk	Low risk	Low risk	Low risk	Some concerns

**Trammell (2017) - Study 3**	Acute self-perceived stress	Effect of assignment to intervention	Some concerns	Low risk	Low risk	Some concerns	Low risk	Some concerns
Trammell (2017) - Study 3	Memory test	Effect of assignment to intervention	Some concerns	Low risk	Low risk	Low risk	Low risk	Some concerns
**Trammell (2019)**	Memory test	Effect of assignment to intervention	Some concerns	Low risk	Low risk	Low risk	Some concerns	Some concerns
Trammell (2019)	Happiness	Effect of assignment to intervention	Some concerns	Low risk	Low risk	Some concerns	Some concerns	Some concerns
Trammell (2019)	Acute self-perceived stress	Effect of assignment to intervention	Some concerns	Low risk	Low risk	Some concerns	Some concerns	Some concerns
Trammell (2019)	Arousal	Effect of assignment to intervention	Some concerns	Low risk	Low risk	Some concerns	Some concerns	Some concerns
**Ward-Griffin et al. (2018)**	Positive and negative affect	Effect of assignment to intervention	Some concerns	Low risk	Low risk	Low risk	Low risk	Some concerns
Ward-Griffin et al. (2018)	Chronic self-perceived stress	Effect of assignment to intervention	Some concerns	Low risk	Low risk	Low risk	Low risk	Some concerns
Ward-Griffin et al. (2018)	Happiness	Effect of assignment to intervention	Some concerns	Low risk	Some concerns	Low risk	Low risk	Some concerns
