# Supplementary material for: Animal-Assisted Interventions Improve Mental, But Not Cognitive or Physiological Health Outcomes of Higher Education Students: a Systematic Review and Meta-analysis
Source: Int J Ment Health Addict. 2022 Nov 15:1–32. Online ahead of print. doi: 10.1007/s11469-022-00945-4 (PMC9666958; doi:10.1007/s11469-022-00945-4)
Supplement: Supplementary file 35 — Supplementary Table S18 (PDF 52 KB) [file 11469_2022_945_MOESM35_ESM.pdf]

Table SXVIII. Quality assessment results for crossover RCTs at the individual outcome level (n(outcomes)=17).

Reference	Outcome	Effect of adhering to intervention?	Randomization process	Bias arising from period and carryover effects	Deviations from intended interventions	Mising outcome data	Measurement of the outcome	Selection of the reported result	Overall Bias
**Barker et al. (2016)**	Acute self-perceived stress	Effect of assignment to intervention	Some concerns	Some concerns	Low risk	Low risk	Some concerns	Low risk	Some concerns
**Crump et al. (2015) - Study I**	Acute self-perceived stress	Effect of assignment to intervention	Some concerns	Some concerns	Low risk	Low risk	Some concerns	Low risk	Some concerns
Crump et al. (2015) - Study I	Arousal	Effect of assignment to intervention	Some concerns	Some concerns	Low risk	Low risk	Some concerns	Low risk	Some concerns
Crump et al. (2015) - Study I	BP	Effect of assignment to intervention	Some concerns	Some concerns	Low risk	Low risk	Low risk	Low risk	Some concerns
Crump et al. (2015) - Study I	HR	Effect of assignment to intervention	Some concerns	Some concerns	Low risk	Low risk	Low risk	Low risk	Some concerns
**Gee et al. (2014)**	HR	Effect of assignment to intervention	Some concerns	Some concerns	Low risk	Low risk	Low risk	Low risk	Some concerns
Gee et al. (2014)	HRV	Effect of assignment to intervention	Some concerns	Some concerns	Low risk	Low risk	Low risk	Low risk	Some concerns
**Gee et al. (2015)**	HR	Effect of assignment to intervention	Some concerns	Some concerns	Low risk	Low risk	Low risk	Low risk	Some concerns
Gee et al. (2015)	HRV	Effect of assignment to intervention	Some concerns	Some concerns	Low risk	Low risk	Low risk	Low risk	Some concerns
Gee et al. (2015)	Memory test	Effect of assignment to intervention	Some concerns	Some concerns	Low risk	Low risk	Low risk	Low risk	Some concerns
**Gee et al. (2019) - Experiment 1**	Happiness	Effect of assignment to intervention	Some concerns	Some concerns	Low risk	Low risk	Some concerns	Low risk	Some concerns
Gee et al. (2019) - Experiment 1	HR	Effect of assignment to intervention	Some concerns	Some concerns	Low risk	Low risk	Low risk	Low risk	Some concerns
Gee et al. (2019) - Experiment 1	HRV	Effect of assignment to intervention	Some concerns	Some concerns	Low risk	Low risk	Low risk	Low risk	Some concerns
**Kobayashi et al. (2017)**	Arousal	Effect of assignment to intervention	Some concerns	Some concerns	Low risk	Low risk	Some concerns	Low risk	Some concerns
**Wilson (1987)**	BP	Effect of assignment to intervention	Some concerns	Some concerns	Low risk	Low risk	Low risk	Low risk	Some concerns
Wilson (1987)	HR	Effect of assignment to intervention	Some concerns	Some concerns	Low risk	Low risk	Low risk	Low risk	Some concerns
Wilson (1987)	Acute and chronic anxiety	Effect of assignment to intervention	Some concerns	Some concerns	Low risk	Low risk	Some concerns	Low risk	Some concerns
